# Neutralization of excessive levels of active TGF-β1 reduces MSC recruitment and differentiation to mitigate peritendinous adhesion

**DOI:** 10.1038/s41413-023-00252-1

**Published:** 2023-05-08

**Authors:** YuSheng Li, Xiao Wang, Bo Hu, Qi Sun, Mei Wan, Andrew Carr, Shen Liu, Xu Cao

**Affiliations:** 1 Department of Orthopedic Surgery, The Johns Hopkins University School of Medicine, Baltimore, MD 21205 USA; 2 Nuffield Department of Orthopedics, Rheumatology and Musculoskeletal Sciences, Botnar Research Centre, University of Oxford, Windmill Road, Oxford, OX3 7LD UK

**Keywords:** Pathogenesis, Bone

## Abstract

Peritendinous adhesion formation (PAF) can substantially limit the range of motion of digits. However, the origin of myofibroblasts in PAF tissues is still unclear. In this study, we found that the concentration of active TGF-β1 and the numbers of macrophages, mesenchymal stromal cells (MSCs), and myofibroblasts in human and mouse adhesion tissues were increased. Furthermore, knockout of TGF-β1 in macrophages or TGF-β1R2 in MSCs inhibited PAF by reducing MSC and myofibroblast infiltration and collagen I and III deposition, respectively. Moreover, we found that MSCs differentiated into myofibroblasts to form adhesion tissues. Systemic injection of the TGF-β–neutralizing antibody 1D11 during the granulation formation stage of PAF significantly reduced the infiltration of MSCs and myofibroblasts and, subsequently, PAF. These results suggest that macrophage-derived TGF-β1 recruits MSCs to form myofibroblasts in peritendinous adhesions. An improved understanding of PAF mechanisms could help identify a potential therapeutic strategy.

## Introduction

In clinical practice, the term “tissue adhesion” is restricted to the abnormal conjunction of anatomical tissues with one another.^1,2^ These are seen most frequently after peritendinous,^3–5^ gynecological,^6,7^ cardiovascular,^8,9^ or abdominal surgery.^10,11^ Among them, peritendinous adhesion formation (PAF) represents a major clinical complication after tendon operation, resulting in nearly $1.3 billion in health care expenditures in the United States in 1994.^12^ Clinical and experimental investigations suggest that PAF occurs in the area named Bunnell’s “no man’s land” or Zone II involving flexor digitorum profundus (FDP) and flexor digitorum superficialis (FDS) tendons, where two tendons glide within a synovial sheath in the fingers.^13^ PAF causes pain, loss of function, and stiffness and greatly limits the range of motion of patients^14,15^ and severely incapacitates people in their daily life. However, the exact mechanism of PAF remains unclear.

The pathology of PAF refers to fibrous strands of internal scar tissue, which are also known as PAF tissues, that are left by the binding of tendons, regardless of injury or intact status, to their surrounding tissues, such as muscles, synovial sheath and FDS.^16,17^ The small number of available therapies in the medical field are limited and of disputed clinical efficacy. The standard treatment for PAF is tenolysis,^18,19^ which is associated with a high recurrence rate and complications, including infection.^20,21^ The current explanation of PAF and its recurrence after tenolysis focuses on the occurrence of PAF tissues outside the tendon and the accumulation of myofibroblasts from tissues surrounding tendons.^13,17,22–25^ However, when we use anti-adhesion membranes to separate PAF tissues, PAF can still be detected on the tendon that is wrapped by these membranes.^26–28^ Therefore, the origin of myofibroblasts in PAF tissues needs further investigation.

PAF is divided into a series of pathological changes, including inflammation (initial stage), granulation formation (early stage) and remodeling (late stage). During the development of PAF, type I and III collagen are overexpressed by the many myofibroblasts present in PAF.^29–32^ Despite the recognized histological features of PAF, little is known about the cell signaling mechanisms that contribute to the pathogenesis of this condition. Macrophages have been reported to be the main immune cell type during the onset of PAF.^33^ These cells have diverse biological functions, which are attributed to two functionally different subpopulations: proinflammatory macrophages and regenerative macrophages.^34–36^ Proinflammatory macrophages are sources of proinflammatory cytokines and induce iNOS production.^37–39^ These cells are important in the elimination of pathogens and initiation of inflammation. In contrast, regenerative macrophages produce anti-inflammatory cytokines and have increased expression of CD206. These cells inhibit inflammation and participate in cell proliferation and matrix deposition.^40–42^ However, the exact cellular relationship between macrophages and myofibroblasts in developing pathological processes is still unknown.

**Scheme 1:**
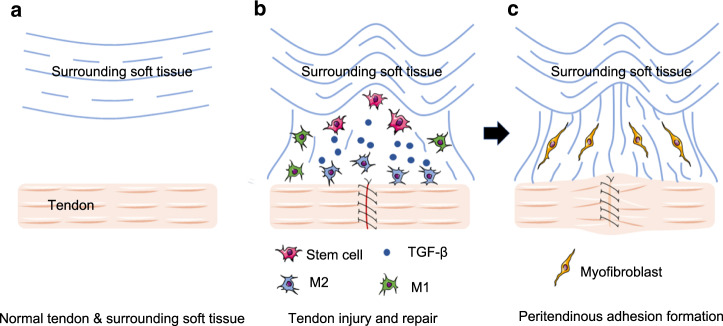
Excessive levels of active TGF-β1 facilitate MSC recruitment and differentiation to mitigate peritendinous adhesion. Normal structure of the tendon and surrounding tissues (**a**). Macrophage-derived TGF-β1 in the accumulation of myofibroblasts outside tendons during PAF (**b**). MSCs gave rise to myofibroblasts and further promoted PAF (**c**)

Previously, we showed that macrophages were a source of transforming growth factor β1 (TGF-β1).^43^ A pioneering observational study showed that TGF-β1 neutralizing antibodies increased the postoperative range of motion in flexor tendon wound healing in rabbits.^44^ However, the underlying mechanism of TGF-β1 in PAF is unknown. We have previously shown that TGF-β1 is activated during tissue remodeling to recruit mesenchymal stromal cells (MSCs) to maintain tissue homeostasis;^45^ furthermore, aberrant activation or excessively high levels of macrophage-derived TGF-β are associated with different diseases,^46^ such as enthesopathy^47^ and heterotopic ossification in the Achilles tendon.^43^ However, whether MSCs recruited by macrophage-derived TGF-β1 can differentiate into myofibroblasts in PAF tissues outside the tendons is also unknown. Therefore, in this study, we investigated the role of macrophage-derived TGF-β1 in the accumulation of myofibroblasts outside the tendons during PAF. We found that macrophages produced TGF-β1 to recruit MSCs to the tendon lesions, which subsequently gave rise to myofibroblasts and PAF. Inhibiting TGF-β signaling attenuated PAF by preventing MSC recruitment and differentiation (Scheme 1).

## Results

### Macrophages are increased in human PAF patients with elevated TGF-β activity

**Fig. 1:**
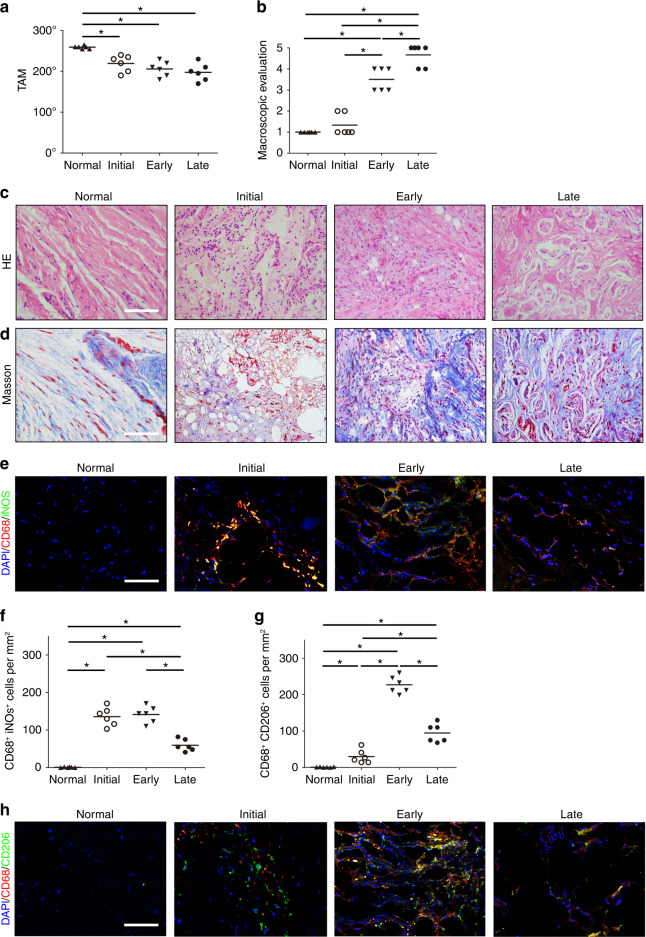
Macrophages are increased in PAF patients. Quantitative analysis of total active motion (TAM) (**a**) and macroscopic adhesion scores (**b**). H&E staining (**c**) and Masson staining (**d**) of normal tissues surrounding the tendons and PAF tissues in the initial, early, and late stages after tendon injury. Scale bar, 200 μm. Immunofluorescent staining of CD68^+^ (red) iNOS^+^ (green) cells (**e**) and quantitative analysis of CD68^+^ iNOS^+^ cells (**f**) per PAF tissue area (mm^2^). Immunofluorescent staining of CD68^+^ (red) CD206^+^ (green) cells (**g**) and quantitative analysis of CD68^+^ CD206^+^ cells (**h**) per PAF tissue area (mm^2^). Blue indicates DAPI staining of nuclei. Scale bar, 200 μm. indicates *P* < 0.05; Normal, normal tissues surrounding tendons. Initial, Early, and Late indicate the time after tendon injury (1–2 days, 8–10 days, and 12–14 weeks, respectively)

We first examined the sum of the range of motion (ROM) at the metacarpophalangeal joints and interphalangeal joints of patients after ruptured tendon repair during the initial stage (1–2 days after injury), early stage (8–10 days after injury) and late stage (12–14 weeks after injury) by assessing total active motion. A decrease in total active motion in repaired hands relative to normal hands was noted (Fig. 1a). The severity of PAF increased from the initial stage to the late stage, as determined by macroscopic evaluation using the adhesion scoring system^26^ (Fig. 1b), indicating adhesion formation around the tendons. Peritendinous adhesion tissues were collected from the same patients. Hematoxylin and eosin (H&E) staining and Masson staining showed abundant inflammatory cell infiltration around the tendon lesions during the initial stage of PAF. The cellular matrix of normal tissues around tendons was oriented in order, but the cellular matrix of peritendinous adhesion tissues was disorganized, and granulation tissues and hypercellular fibrotic tissues were detected during the early and late stages of PAF (Fig. 1c, d). The biological functions of macrophages are attributed to 2 functionally different subpopulations: classically activated (M1) macrophages and selectively activated (M2) macrophages.^34,36,41^ M1 macrophages mainly enhance inflammation,^37–39^ whereas M2 macrophages typically inhibit inflammation and promote cell proliferation and matrix deposition.^40–42^ To identify the subtypes of macrophages in peritendinous tissues, we stained for iNOS and CD68 to label M1 monocytes/macrophages and for CD206 and CD68 to label M2 monocytes/macrophages.^48,49^ The number of CD68^+^ iNOS^+^ M1 monocytes/macrophages in PAF tissues increased significantly during the initial stage, plateaued during the early stage, and decreased dramatically during the late stage (Fig. 1e, f). Conversely, the number of CD68^+^ CD206^+^ M2 monocytes/macrophages in PAF tissues was elevated during the initial stage, followed by a sharp, nearly 10-fold increase during the early stage (Fig. 1g, h). Furthermore, M2 macrophages were still detected during the late stage, while few M1 macrophages were observed.

**Fig. 2:**
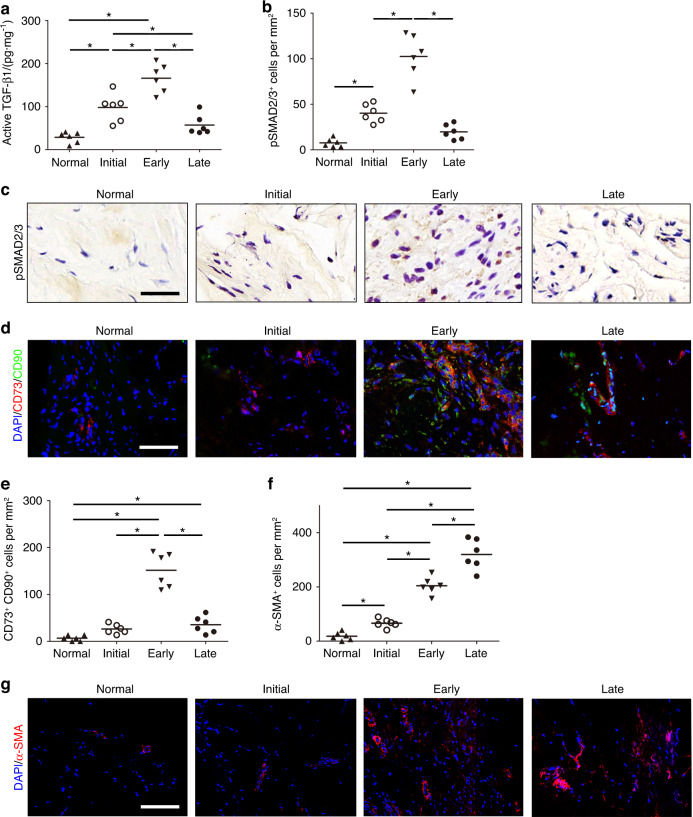
TGF-β activity is elevated in PAF patients. Active TGF-β1 (**a**). Quantitative analysis (**b**) and immunohistological staining (**c**) of pSmad2/3^+^ cells (brown) per PAF tissue area (mm^2^). Scale bar, 400 μm. Immunofluorescence staining (**d**) and the number (**e**) of CD73^+^ (red) and CD90^+^ (green) cells per PAF tissue area (mm^2^). Immunofluorescence staining (**f**) and the number (**g**) of α-SMA^+^ (red) cells per PAF tissue area (mm^2^). Blue indicates DAPI staining of nuclei. Scale bar, 200 μm. * indicates *P* < 0.05; Normal, normal tissues surrounding tendons. Initial, Early, and Late indicate the time after tendon injury (1–2 days, 8–10 days, and 12–14 weeks, respectively)

We then measured the levels of active TGF-β1 in PAF tissues. The concentrations of active TGF-β1 during the initial and early stages were significantly higher than those of healthy controls and those in the late stage (Fig. 2a), and the highest level occurred during the early stage. We used immunohistological staining of phosphorylated Smad2/3 (pSmad2/3^+^), which is a TGF-β downstream signaling transducer, to examine the involvement of active TGF-β in PAF tissues. We found that the number of pSmad2/3^+^ cells increased significantly during the initial and early stages and decreased during the late stage (Fig. 2b, c), whereas there were no significant differences in the numbers of cells that were positive for pSmad1/5/8, a bone morphogenetic protein 2 (BMP-2) downstream signaling transducer among the different stages (Fig. S1A, B). Importantly, the activation pattern of latent TGF-β1 was similar to that of M2 macrophages but not M1 macrophages, suggesting the involvement of M2 macrophages in TGF-β1 activation during PAF.

Immunofluorescent staining of CD73^+^ CD90^+^ MSC-like cells^50,51^ revealed that the number of MSC-like cells in PAF tissues was higher than that in healthy tissues and that it peaked during the early stage (Fig. 2d, e). Immunofluorescent staining of myofibroblasts using alpha-smooth muscle actin (α-SMA) as a marker^52,53^ showed that α-SMA^+^ myofibroblasts accumulated in peritendinous tissues during PAF from the initial to the late stage, and thus, the highest number of myofibroblasts was seen during the late stage (Fig. 2f, g). The deposition of collagen I and collagen III fibers in PAF tissues also increased during the formation of peritendinous adhesions (Fig. S1C–F). Taken together, these results reveal that macrophages are increased in the PAF tissues of humans, and there are elevated levels of active TGF-β, MSC-like cells, and myofibroblasts in the PAF microenvironment.

### Macrophages and active TGF-β levels are increased in mouse PAF tissues

**Fig. 3:**
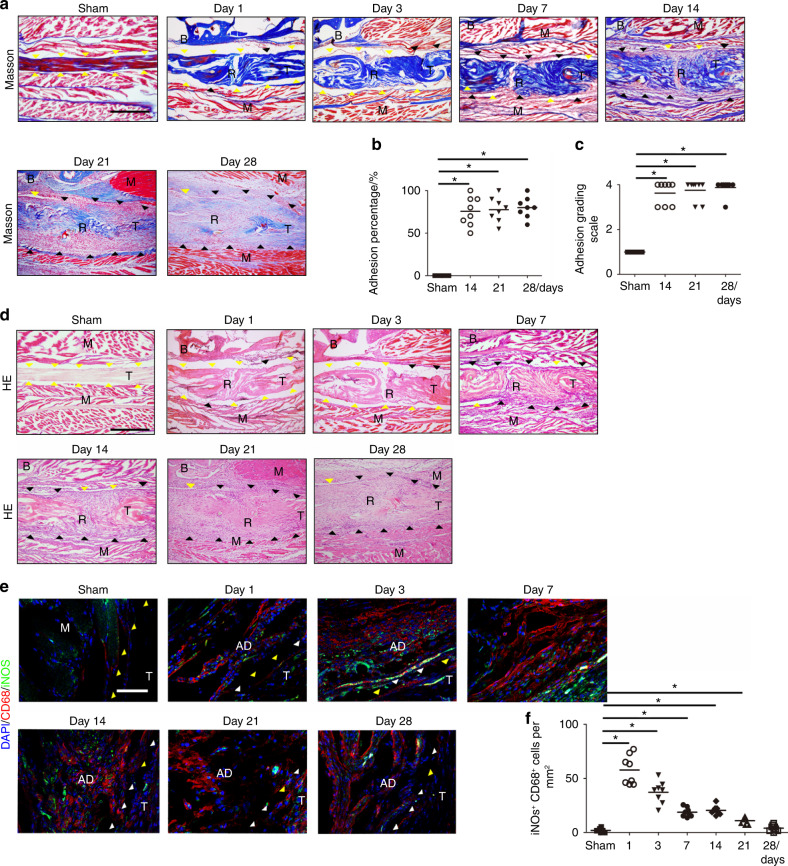
M1 monocyte/macrophage levels are increased in mouse PAF tissues. Masson staining (**a**) and quantitative analysis of the space between the tendon and surrounding tissues (**b**) at days 14, 21 and 28 after tendon injury. Histological adhesion scores (**c**) and H&E staining (**d**) of normal tissues around tendons and PAF tissues on postoperative Days 1, 3, 7, 14, 21, and 28. Yellow arrowheads indicate the space between the tendon and surrounding tissues. Black arrowheads indicate the space occupied by adhesion tissues. Scale bar, 1 mm. Immunofluorescent staining of CD68^+^ (red) iNOS^+^ (green) cells (**e**) and quantitative analysis of CD68^+^ iNOS^+^ cells (**f**) per PAF tissue area (mm^2^). White arrowheads indicate the space between the tendon and surrounding tissues. Yellow arrowheads indicate the space occupied by adhesion tissues. Scale bar, 400 μm. * indicates *P* < 0.05. B, bone; D, day; M, muscle; R, repaired site; ROM, range of motion; T, tendon

To investigate the pathomechanism of PAF, we created a mouse model in which we transversally transected and repaired the flexor digitorum longus (FDL) tendon using sutures in a modified Kessler pattern as previously described.^54,55^ Masson staining of PAF tissues showed extensive peritendinous adhesion tissue formation in the PAF model on postoperative Days 7 to 28, which was significantly greater than that in the sham operative controls according to quantitative analysis of the adhesion percentage (Fig. 3a, b). Furthermore, the area of PAF tissue invasion in the repaired tendons increased. We also found increased deposition of collagen I and collagen III in PAF mice on postoperative Day 7, further suggesting the formation of adhesion tissues (Fig. S2A–D).

Inflammatory cell infiltration was observed in PAF tissues on postoperative Days 1 to 7. We observed granulation tissues with disorganized cellular matrix and hypercellular fibrotic tissues around the tendons compared with the oriented cellular matrix and muscles around normal tendons (Fig. 3c, d). Peritendinous adhesions were first present at the repair sites on postoperative Day 7 and continued to form until Day 28 (Fig. 3d). The histological adhesion scores were significantly higher in the PAF model than in the sham controls, indicating severe peritendinous adhesions around the tendon lesions (Fig. 3c).

**Fig. 4:**
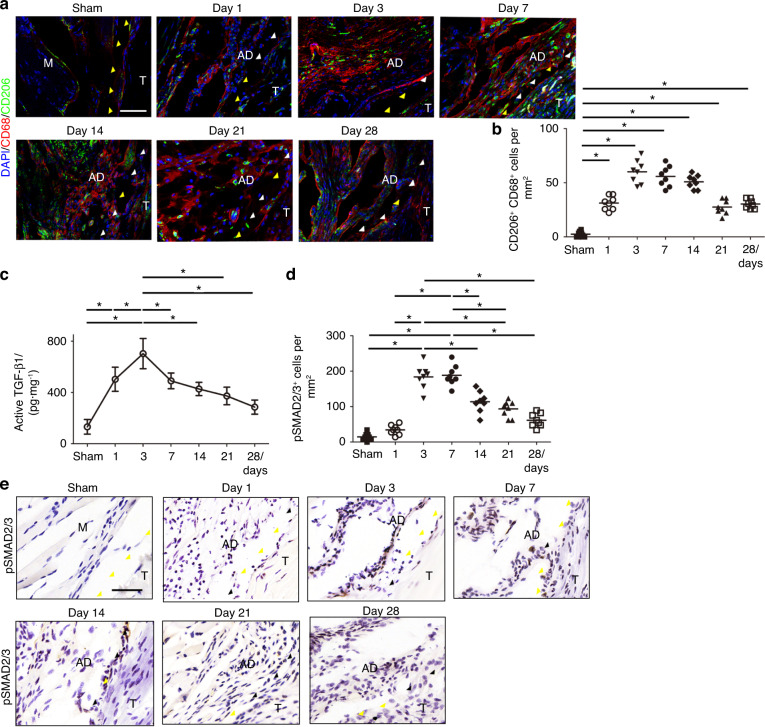
M2 monocytes/macrophages and active TGF-β levels are increased in mouse PAF tissues. Immunofluorescent staining of CD68^+^ (red) CD206^+^ (green) cells (**a**) and quantitative analysis of CD68^+^ CD206^+^ cells (**b**) per PAF tissue area (mm^2^). Blue indicates DAPI staining of nuclei. White arrowheads indicate the space between the tendon and surrounding tissues. Yellow arrowheads indicate the space occupied by adhesion tissues. Scale bar, 200 μm. Active TGF-β1 (**c**). Quantitative analysis (**d**) and immunohistological staining (**e**) of pSmad2/3^+^ cells (brown) per PAF tissue area (mm^2^). White arrowheads indicate the space between the tendon and surrounding tissues. Yellow arrowheads indicate the space occupied by adhesion tissues. Scale bar, 400 μm. * indicates *P* < 0.05. B, bone; D, day; M, muscle; R, repaired site; ROM, range of motion; T, tendon

Immunofluorescent staining of CD68^+^ iNOS^+^ M1 monocytes/macrophages (Fig. 3e) revealed that the cell numbers were significantly increased in PAF tissues on postoperative Day 1 and gradually decreased to baseline on Day 28, when no significant differences were observed compared with the sham controls (Fig. 3f). Conversely, the number of CD68^+^ CD206^+^ M2 monocytes/macrophages was significantly increased on postoperative Day 1, peaking on Day 3, plateauing on Day 7, and decreasing from Day 14. M2 macrophage numbers were still significantly higher in PAF tissues than in sham controls on postoperative Days 14, 21, and 28 (Fig. 4a, b).

The level of active TGF-β1 in mouse PAF tissues increased on postoperative Days 1 to 3 before decreasing from Days 7 to 28 (Fig. 4c). Immunohistochemical staining of pSmad2/3 showed that the number of pSmad2/3^+^ cells peaked on Day 7 and gradually decreased on postoperative Day 14 (Fig. 4d, e). Similar to our observation in human specimens, the expression of active TGF-β1 was consistent with M2 macrophages but not M1 macrophages in the mouse PAF model, suggesting that M2 macrophages were the sources of active TGF-β1. There were no significant differences in the expression of pSmad1/5/8 in mouse adhesion tissues (Fig. S2E, F).

**Fig. 5:**
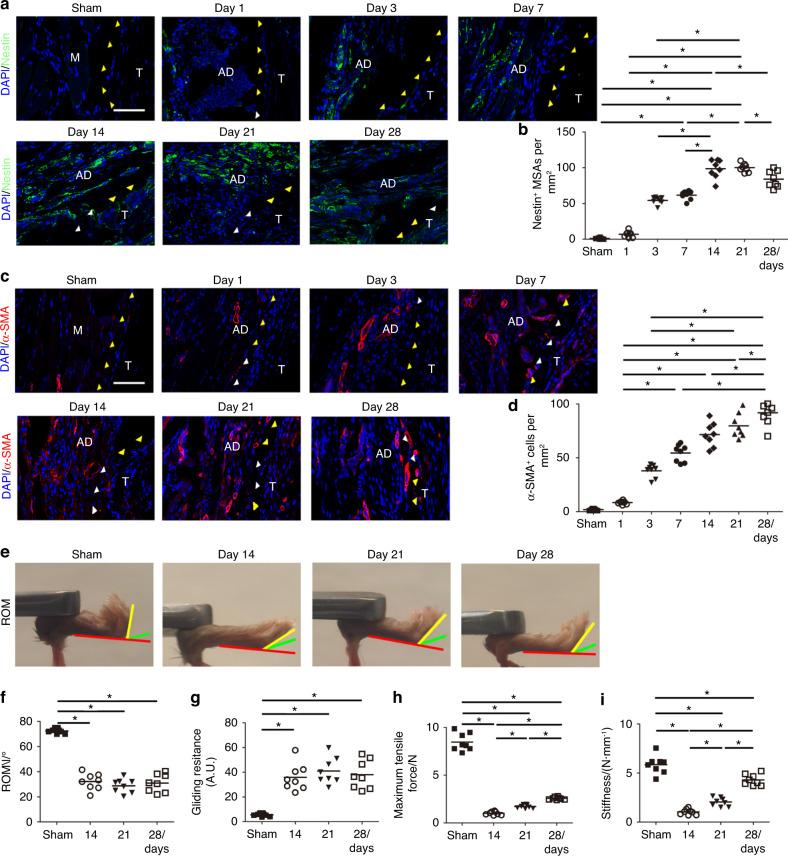
Immunofluorescence staining (**a**) and the number (**b**) of Nestin^+^ (green) cells per PAF tissue area (mm^2^) on postoperative Days 1, 3, 7, 14, 21, and 28. Immunofluorescence staining (**c**) and the number (**d**) of α-SMA^+^ (red) cells per PAF tissue area (mm^2^) at 1, 3, 7, 14, 21, and 28 days after tendon injury. White arrowheads indicate the space between the tendon and surrounding tissues. Yellow arrowheads indicate the space occupied by adhesion tissues. Blue indicates DAPI staining of nuclei. Scale bar, 200 μm. Investigation (**e**) and quantitative analysis (**f**) of ROM at 14 days, 21 days, and 28 days. Quantitative analysis of gliding resistance (**g**), maximum tensile force (**h**), and stiffness (**i**) of repaired tendons. * indicates *P* < 0.05. B, bone; D, day; M, muscle; R, repaired site; ROM, range of motion; T, tendon

We found that Nestin^+^ MSCs^56–58^ were increased significantly in PAF tissues on postoperative Days 1 to 14 and peaked on Day 14 and decreasing on Day 28 (Fig. 5a, b). Immunofluorescent staining of α-SMA showed that the number of α-SMA^+^ cells increased by nearly 16 times on postoperative Day 1 to Day 28 (Fig. 5c, d). To examine the effect of adhesion tissues on tendon gliding in PAF mice, we tested the range of motion (ROM) of metacarpophalangeal joints and gliding resistance^59,60^ on postoperative Days 14, 21, and 28, when PAF tissues were being formed. A significant decrease in the ROM was detected in PAF mice compared with the sham controls (Fig. 5e, f). Gliding resistance was significantly higher in the adhesion model than the controls (Fig. 5g). Biomechanical analysis showed significantly less maximum tensile force and stiffness in repaired tendons than in intact tendons (Fig. 5h, i), suggesting inferior mechanical properties of repaired tendons with adhesion. Overall, the PAF mouse model resembled human PAF specimens and had a similar mechanism, indicating that excessive levels of active TGF-β1 and macrophages contribute to the pathogenesis of PAF.

### Macrophage-derived TGF-β1 triggers PAF

**Fig. 6:**
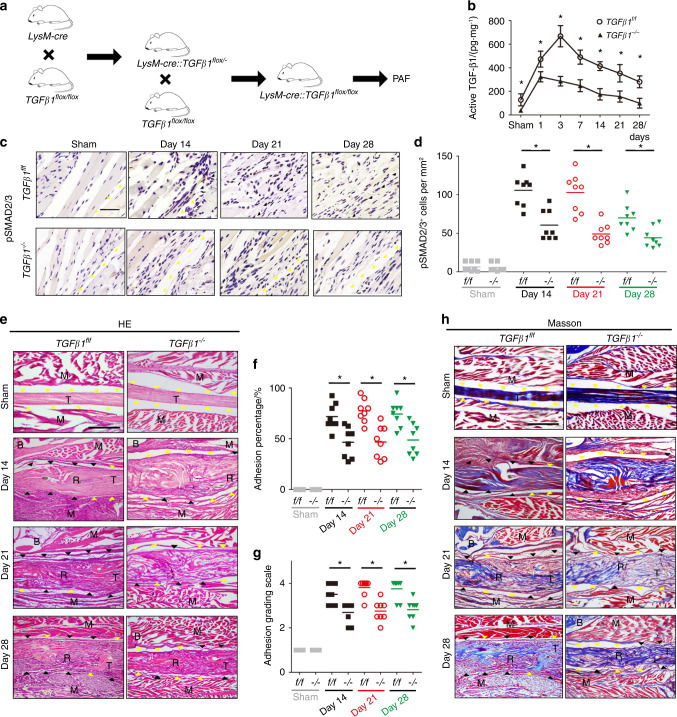
Knockout of TGF-β1 in Lysm^+^ cells reduces peritendinous adhesions. *LysM-cre*::*TGF-β1*^*flox/flox*^ (*TGF-β1*^*–/–*^) mice were generated by crossing *LysM-cre* mice with *TGF-β1*^*flox/flox*^ (*TGF-β1*^*f/f*^) mice (**a**). Active TGF-β1 (**b**) in normal tissues surrounding tendons and PAF tissues at 1, 3, 7, 14, 21, and 28 days in *LysM-cre*::*TGF-β1*^*flox/flox*^ mice. Immunohistological staining (**c**) and quantitative analysis (**d**) of pSmad2/3^+^ cells (brown) per PAF tissue area (mm^2^). Yellow arrowheads indicate the space between the tendon and surrounding tissues. Black arrowheads indicate the space occupied by adhesion tissues. Scale bar, 400 μm. H&E staining (**e**) and histological adhesion scores (**f**) of PAF tissues on sham and postoperative Days 14, 21, and 28. Masson staining (**h**) and quantitative analysis (**g**) of the space between the tendon and its tissues. Yellow arrowheads indicate the space between the tendon and surrounding tissues. Black arrowheads indicate the space occupied by adhesion tissues. Scale bar, 1 mm. indicates *P* < 0.05. *TGF-β1*^*flox/flox*^ mice, *TGF-β1*^*f/f*^*(f/f)*. B bone; D, day; M, muscle; R, repaired site; T, tendon

We observed the infiltration of macrophages and high levels of active TGF-β1 during the initial and early stages of PAF in adhesion tissues from both human subjects and PAF mice. This prompted us to investigate whether macrophages produce TGF-β1, which triggers PAF. We generated *LysM-cre*::*TGF-β1*^*flox/flox*^ (*TGF-β1*^*–/–*^) mice, in which macrophages/granulocytes^61^ do not produce TGF-β1, by breeding *LysM-cre* mice with *TGF-β1*^*flox/flox*^ (*TGF-β1*^*f/f*^) mice (Fig. 6a). We first verified that TGF-β1 was successfully deleted in macrophages in the PAF tissues of *TGF-β1*^*–/–*^ mice. We found that active TGF-β1 expression in PAF tissues was significantly decreased in *TGF-β1*^*–/–*^ mice compared with *TGF-β1*^*f/f*^ mice (Fig. 6b). Immunohistological staining of pSmad2/3^+^ (Fig. 6c) showed that there were significantly fewer pSmad2/3^+^ cells in *TGF-β1*^*–/–*^ mice than in controls (Fig. 6d). Taken together, these results validated that TGF-β1 secretion by macrophages was mitigated in *TGF-β1*^*–/–*^ mice.

**Fig. 7:**
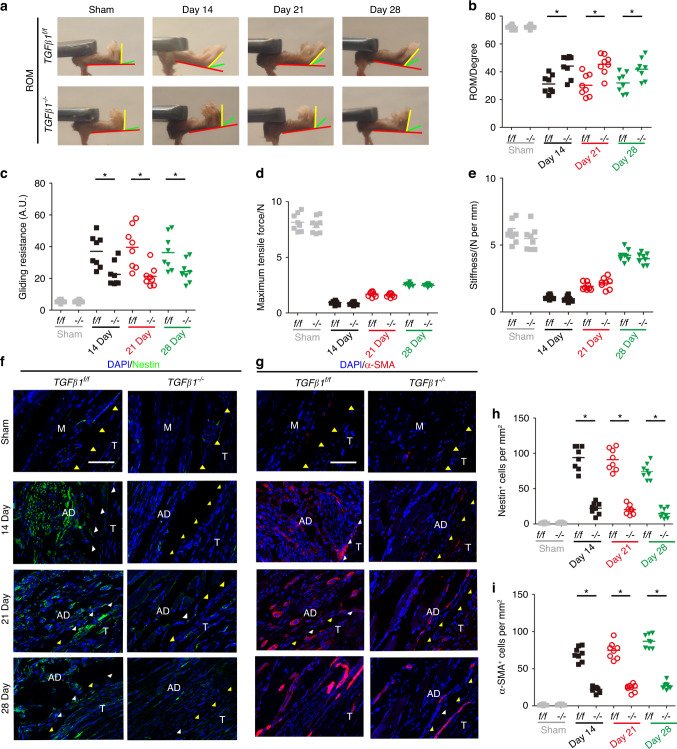
Knockout of TGF-β1 in Lysm^+^ cells attenuates peritendinous adhesions and reduces Nestin^+^ and α-SMA^+^ cells. Investigation (**a**) and quantitative analysis (**b**) of the ROM. Quantitative analysis of the gliding resistance (**c**), maximum tensile force (**d**), and stiffness (**e**) of repaired tendons in *LysM-cre*::*TGF-β1*^*flox/flox*^ mice (*TGF-β1*^*–/–*^). Immunofluorescence staining of Nestin^+^ (green) cells (**f**) and α-SMA^+^ (red) cells (**g**) per PAF tissue area (mm^2^) and the number of Nestin^+^ (green) cells (H) and α-SMA^+^ (red) cells (**i**) per PAF tissue area (mm^2^) in the sham and postoperative Days 14, 21, and 28 groups. Scale bar, 200 μm. Yellow arrowheads indicate the space between the tendon and surrounding tissues. White arrowheads indicate the space occupied by adhesion tissues. Blue indicates DAPI staining of nuclei. Scale bar, 200 μm. * indicates *P* < 0.05. *TGF-β1*^*flox/flox*^ mice, *TGF-β1*^*f/f*^*(f/f)*. B bone; D, day; M, muscle; R, repaired site; ROM, range of motion; T, tendon

H&E staining of adhesion tissues showed significantly less granulation tissue formation in *TGF-β1*^*–/–*^ PAF mice than in *TGF-β1*^*f/f*^ PAF mice (Fig. 6e). Moreover, the surfaces of repaired tendons were smoother, and the space between the tendon and surrounding tissue was larger in *TGF-β1*^*–/–*^ mice than in *TGF-β1*^*f/f*^ mice. The histological adhesion scores of *TGF-β1*^*–/–*^ mice were significantly lower than those of *TGF-β1*^*f/f*^ mice at various time points (Fig. 6f). In addition, Masson staining of PAF tissues revealed that adhesion tissues were significantly decreased, and there was a lower adhesion percentage in *TGF-β1*^*–/–*^ mice than in wild-type controls (Fig. 6g, h). Collagen I and collagen III deposition (Fig. S3A, B) and quantitative analysis (Fig. S3C, D) showed significantly less integral optical density in *TGF-β1*^*–/–*^ mice than in *TGF-β1*^*f/f*^ mice. These results suggest that less adhesion tissue formed after TGF-β1 production was inhibited in macrophages. We next performed mechanical testing and found that the ROM was significantly increased and the gliding distance was reduced in *TGF-β1*^*–/–*^ mice compared with *TGF-β1*^*f/f*^ mice (Fig. 7a–c), further validating the attenuation of adhesion after TGF-β1 was knocked out in macrophages. There were no statistically significant differences in the maximum tensile force or stiffness of repaired tendons between *TGF-β1*^*–/–*^ and *TGF-β1*^*f/f*^ PAF mice, which indicates that knocking out TGF-β1 in macrophages did not alter the mechanical properties of repaired tendons (Fig. 7d, e). Immunostaining of Nestin and α-SMA revealed that the numbers of Nestin^+^ MSCs and α-SMA^+^ myofibroblasts were significantly lower in *TGF-β1*^*–/–*^PAF mice than in *TGF-β1*^*f/f*^ mice at the respective time points (Fig. 7f–i). Overall, inhibiting TGF-β1 secretion by macrophages reduced adhesion and the numbers of MSCs and myofibroblasts, indicating that TGF-β1 produced by macrophages initiates PAF.

### Type 2 TGF-β receptor (TGF-βR2) knockout in Nestin^+^ MSCs attenuates PAF

**Fig. 8:**
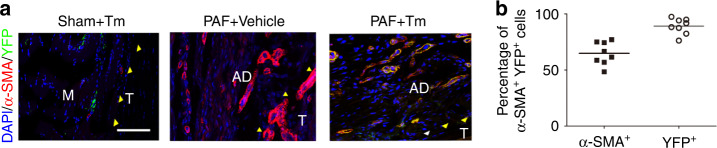
Nestin^+^ cells differentiate into α-SMA^+^ cells in PAF tissues. Alpha-SMA^+^ cells (red) and YFP^+^ cells (green) in normal tissues surrounding tendons and PAF tissues of *Nestin-creER*^*T2*^*::R26R-EYFP* mice injected with tamoxifen on postoperative Day 28 (28 D) (**a**). Quantitative analysis of α-SMA^+^ (red) cells and YFP^+^ (green) cells (**b**). Yellow arrowheads indicate the space between the tendon and surrounding tissues. White arrowheads indicate the space occupied by adhesion tissues. Blue indicates DAPI staining of nuclei. Scale bar, 200 μm. B, bone; D, day; M, muscle; R, repaired site; T, tendon. * indicates *P* < 0.05

To understand the function of Nestin^+^ MSCs during PAF progression, Nestin^+^ MSCs were investigated using *Nestin-creER*^*T2*^*::R26R-EYFP* mice by breeding *Nestin-creER*^*T2*^ mice with *R26R-EYFP* mice, in which Nestin^+^ lineage cells were labeled with yellow fluorescent protein (YFP) in response to tamoxifen injection. The vessels indicated by multiple α-SMA^+^ cells in a hollow tube shape were excluded when calculating α-SMA^+^ myofibroblasts. Immunofluorescent staining of α-SMA and YFP revealed that α-SMA^+^ YFP^+^ cells in fusiform and circular shapes were ~65% of α-SMA^+^ myofibroblasts in PAF tissues, whereas few α-SMA^+^ YFP^+^ cells were observed in the sham controls, suggesting that Nestin^+^ MSCs gave rise to α-SMA^+^ cells during adhesion formation (Fig. 8a, b).

**Fig. 9:**
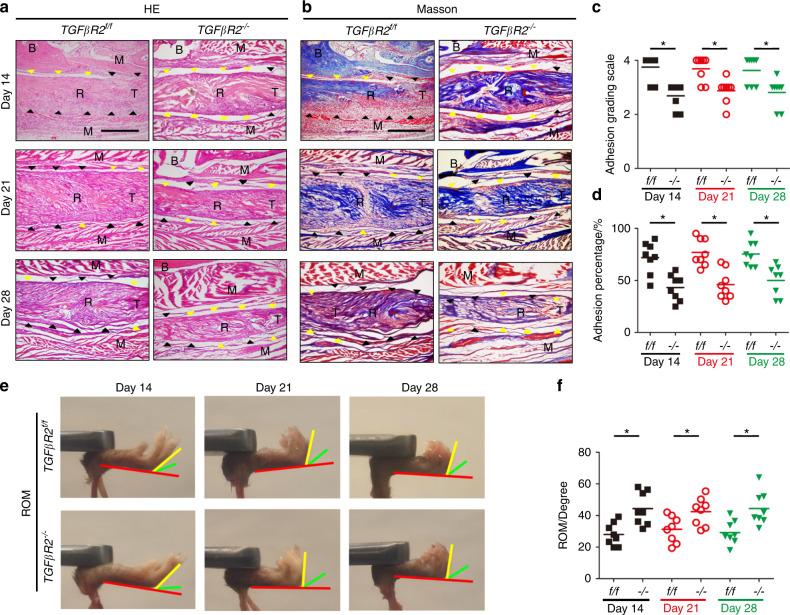
Deletion of TGFβR2 in Nestin^+^ cells attenuates peritendinous adhesion. H&E staining (**a**) and Masson staining (**b**) of PAF tissues and the space between the tendon and its tissues on postoperative Days 14, 21, and 28. Scale bar, 1 mm. Yellow arrowheads indicate the space between the tendon and surrounding tissues. Black arrowheads indicate the space occupied by adhesion tissues. Quantitative analysis of PAF tissues (**c**) and the space between the tendon and its tissues (**d**). Investigation (**e**) and quantitative analysis (F) of ROM. Scale bar, 200 μm. B, bone; D, day; M, muscle; R, repaired site; ROM, range of motion; T, tendon. * indicates *P* < 0.05

**Fig. 10:**
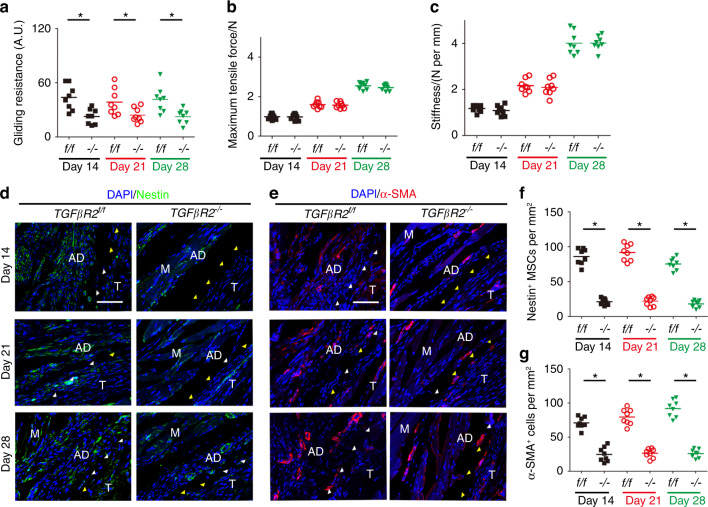
Deletion of TGFβR2 in Nestin^+^ cells reduces Nestin^+^ and α-SMA^+^ cells in PAF tissues. Quantitative analysis of the gliding resistance (**a**), maximum tensile force (**b**), and stiffness (**c**) of repaired tendons in *Nestin-cre ER*^*T2*^::*TGFβR2*^*flox/flox*^ mice (*TGFβR2*^*–/–*^). Immunofluorescence staining of Nestin^+^ (green) cells (**d**) and their number (**f**) at 14 days, 21 days, and 28 days per PAF tissue area (mm^2^). Immunofluorescence staining of α-SMA^+^ (red) cells (**e**) and their number (**g**) at 14 days, 21 days, and 28 days per PAF tissue area (mm^2^). Yellow arrowheads indicate the space between the tendon and surrounding tissues. White arrowheads indicate the space occupied by adhesion tissues. Blue indicates DAPI staining of nuclei. Scale bar, 200 μm. B, bone; D, day; M, muscle; R, repaired site; T, tendon. * indicates *P* < 0.05

We previously demonstrated that active TGF-β1 could recruit stromal cells to remodeling sites.^45,62^ Thus, we further investigated whether Nestin^+^ MSCs were the effector cells of active TGF-β1 during PAF by generating tamoxifen-inducible *Nestin-creER*^*T2*^::*TGFβR2*^*flox/flox*^ mice by breeding *Nestin-creER*^*T2*^ mice with *TGFβR2*^*flox/flox*^ mice. PAF tissues were analyzed by H&E and Masson staining and showed significant decreases in granulation tissues, histological adhesion scores and adhesion percentages in *TGFβR2*^*–/–*^ mice relative to wild-type controls (Fig. 9a–d, Fig. S4A–C) and reduced deposition of collagen I and collagen III by immunostaining (Fig. S5A–D), suggesting the mitigation of PAF in *TGFβR2*^*–/–*^ mice. Increased ROM and decreased gliding resistance were also noted in *TGFβR2*^*–/–*^ mice compared with wild-type controls, indicating improved mechanical properties in *TGFβR2*^*–/–*^ mice (Figs. 9e, f, 10a). No statistically significant differences in maximum tensile force or stiffness were observed between *TGFβR2*^*–/–*^ and *TGFβR2*^*f/f*^ mice, indicating no changes in the mechanical properties of repaired tendons in *TGFβR2*^*–/–*^ mice (Fig. 10b, c). The numbers of Nestin^+^ MSCs (Fig. 10d, f) and α-SMA^+^ myofibroblasts (Fig. 10e, g) in *TGFβR2*^*–/–*^ mice were significantly lower than those in *TGFβR2*^*f/f*^ mice, suggesting that Nestin^+^ MSCs with defects in TGF-β type 2 receptors failed to be recruited to the lesion sites. Taken together, these results reveal that the deletion of *TGFβR2* in Nestin^+^ MSCs can reduce adhesion formation by inhibiting the recruitment of Nestin^+^ MSCs and their further differentiation into α-SMA^+^ myofibroblasts.

### Systemic injection of the TGF-β neutralizing antibody (1D11) attenuates the progression of PAF

**Fig. 11:**
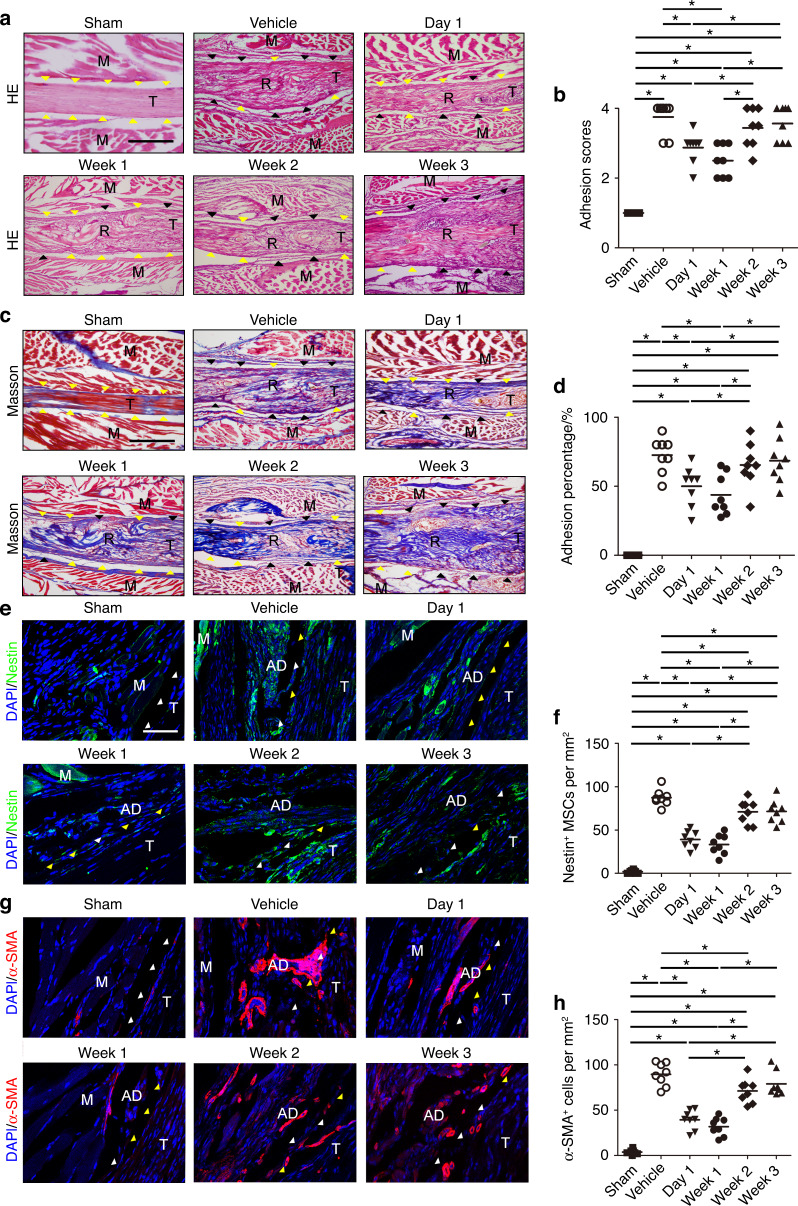
Systemic injection of a TGF-β1 neutralizing antibody attenuates peritendinous adhesion formation. Mice were treated with vehicle 3 times per week for 4 weeks or 5 mg·kg^−1^ body weight TGF-β1 neutralizing antibody (1D11) three times per week on postoperative Day 1, week 1, week 2, or week 3 until euthanasia at week 4. H&E staining (**a**) and histological adhesion scores (**b**) of PAF tissues on postoperative Day 28 (28 D). Scale bar, 1 mm. Masson staining (**c**) and quantitative analysis (**d**) of the space between the tendon and its tissues. Yellow arrowheads indicate the space between the tendon and surrounding tissues. Black arrowheads indicate the space occupied by adhesion tissues. Scale bar, 1 mm. Immunofluorescence staining (**e**) and the number (**f**) of Nestin^+^ (green) cells per PAF tissue area (mm^2^) and immunofluorescence staining (**g**) and the number (H) of α-SMA^+^ (red) cells per PAF tissue area (mm^2^). Yellow arrowheads indicate the space between the tendon and surrounding tissues. White arrowheads indicate the space occupied by adhesion tissues. Blue indicates DAPI staining of nuclei. Scale bar, 200 μm. B, bone; AD, adhesion tissues; M, muscle; R, repaired site; T, tendon. * indicates *P* < 0.05

To investigate the blockade of TGF-β signaling as a potential treatment, we examined the effect of a TGF-β1 neutralizing antibody on PAF at different time points. Briefly, the mice were treated with the TGF-β1 neutralizing antibody 1D11 (5 mg·kg^−1^ body weight) 3 times per week on postoperative Day 1 (Ab 0–1 W), Week 1 (Ab 1–2 W), Week 2 (Ab 2–3 W), or Week 3 (Ab 3–4 W) for 1 week or with vehicle 3 times per week for 4 weeks. 1D11- and vehicle-treated mice were euthanized 4 weeks after surgery. Fewer granulation tissues and histological adhesion scores were observed in PAF mice after 1D11 injection in the Ab 0–1 W and Ab 1–2 W groups relative to vehicle-treated mice and mice with 1D11 injection in the Ab 2–3 W and Ab 3–4 W groups (Fig. 11a–d). Although no significant difference was observed between the Ab 0–1 W and Ab 1–2 W groups, fewer PAF tissues were observed in the Ab 2–3 W group than in the Ab 1–2 W group. No significant decrease in PAF tissues was noted in the Ab 2–3 W and Ab 3–4 W groups relative to vehicle-treated mice. Similarly, the surfaces of repaired tendons were clear in the Ab 0–1 W and Ab 1–2 W groups, and there was a decrease in the adhesion percentage compared with that in vehicle-treated controls. In contrast, PAF tissues occupied the tendon surface in the Ab 2–3 W and Ab 3–4 W groups and the vehicle-treated group. Clusters of Nestin^+^ MSCs and the numbers of α-SMA^+^ cells were significantly decreased in the Ab 0–1 W and Ab 1–2 W groups compared with the vehicle-treated group and Ab 2–3 W and Ab 3–4 W groups (Fig. 11e–h). Mice treated with 1D11 in the Ab 0–1 W and Ab 1–2 W groups showed less collagen I and collagen III deposition than those in the Ab 2–3 W and Ab 3-4 W and vehicle-treated groups (Fig. S6A–D). Significant improvements in ROM and gliding resistance were observed in the Ab 0–1 W and Ab 1–2 W groups compared with the vehicle-treated controls but not in the Ab 2–3 W and Ab 3–4 W groups (Fig. S7A to C). No significant differences were observed in the maximum tensile force and stiffness of repaired tendons among the different treatment groups or the vehicle-treated group (Fig. S7D, E). Our results reveal that systemic injection of the TGF-β1 neutralizing antibody from the first 2 weeks after the operation when TGF-β was elevated could attenuate the progression of PAF by reducing Nestin^+^ MSC recruitment and their further differentiation to α-SMA^+^ myofibroblasts.

## Discussion

Peritendinous adhesion formation between the tendon and surrounding tissues involves a series of pathological changes, including inflammation, granulation formation, and remodeling.^29^ Throughout all three overlapping stages of PAF, macrophages have been reported to play an important role as the dominant immune cell type.^33^ Macrophages have 2 distinct phenotypic states: proinflammatory M1 macrophages and regenerative M2 macrophages. Consistent with previous studies,^33,63,64^ we found macrophage infiltration during the early stage, accumulation during the early stage, and a slight decrease during the late stage in a mouse model of PAF. Most importantly, we showed for the first time that M1 and M2 macrophages had distinct expression patterns; M1 macrophages peaked during the initial stage, and M2 macrophages peaked predominantly during the early stage. This finding suggests a phonotypic transition from M1 to M2 during the pathogenesis of PAF. Macrophages have been reported to be the major sources of active TGF-β1 during fibrosis.^65,66^ In this study, we found that active TGF-β1 levels and macrophage numbers increased during the formation of adhesion tissues. Interestingly, the expression pattern of active TGF-β1 resembled that of M2 macrophages but not M1 macrophages, indicating that M2 macrophages may produce TGF-β1 during PAF. This finding was consistent with previous studies.^67,68^ However, further mechanistic studies could be performed to determine the detailed roles of M1 and M2 macrophages in PAF and are needed to explore the positive and negative effects more thoroughly, preferably using RNA analysis of TGF-β1 expression in M1 and M2 macrophages and specific gene-modified mouse models for validation. More importantly, specific knockout of TGF-β1 in macrophages significantly reduced adhesion formation, verifying that macrophage-derived TGF-β1 initiated PAF.

Collagen I and III are produced by myofibroblasts and have been reported to be the major extracellular matrix components of peritendinous adhesion tissues.^30,69,70^ However, the origin of myofibroblasts is still unknown. In our study, we found that MSCs were significantly increased in the injured site around the repaired tendons during the early stage of adhesion formation in PAF patients and the mouse model. Therefore, we used a lineage tracing method to determine the progeny produced by MSCs during PAF and found that most MSCs gave rise to myofibroblasts. TGF-β signaling is involved in the migration and differentiation of MSCs and the regulation of collagen formation and tissue remodeling through the activation of its canonical pathway and Smad2/3 phosphorylation.^45,71,72^ We previously demonstrated that TGF-β1 could recruit MSCs to injury sites to induce enthesopathy and heterotopic ossification in tendons.^43,47^ In this study, we found a similar mechanism in PAF, and excessive levels of active TGF-β1 recruited MSCs to the adhesion sites and facilitated their differentiation into myofibroblasts. The deletion of the type 2 TGF-β receptor from MSCs or systemic injection of a TGF-β neutralizing antibody during the proliferation stage reduced collagen I and collagen III deposition and improved the ROM, attenuating adhesion tissue formation and further validating TGF-β1 as the driving force of PAF.

In the present study, macrophages reliably induced PAF and increased active TGF-β levels throughout PAF progression. However, there is no direct in vivo evidence to show that M2 but not M1 macrophages are the major sources of TGF-β1 in PAF. Although we found no adverse effects of the systemic use of the TGF-β neutralizing antibody in this study, there are concerns about its safety during long-term use in humans. There was a statistically significant decrease in active TGFβ1 in *TGF-β1*^*–/–*^ mice, indicating that macrophages are the main sources of TGFβ1. However, we cannot exclude other sources of TGFβ1, including the injured tendons, since the TGFβ1 levels were slightly higher in PAF *TGF-β1*^*–/–*^ mice than in sham-operated mice. This finding also indicates that other types of cells participate in adhesion formation. In addition, the effect of MSCs expressing other types of cellular markers in PAF needs further analysis. In this study, we reported that inhibiting TGF-β activity could mitigate adhesion tissue formation. Therefore, inhibiting TGF-β signaling could be a strategy for preventing recurrent PAF and associated disability.

## Methods

### Human subjects

This study was carried out by the review board of Johns Hopkins University (Baltimore, Maryland, USA) and the Shanghai Sixth People’s Hospital (Shanghai, China). The human samples were excised pathological tissue, and signed written informed consent was provided for use in this study. Human tissues around the tendon were collected from 24 patients (15 men and 9 women; age range, 22–46 years) including 6 cases in each of the 3 different stages of PAF and 6 normal cases. We categorized the specimens as normal (i.e., amputated fingers after injury), initial stage (1–2 days after tendon injury), early stage (8–10 days after tendon injury) or late stage (12 weeks after tendon injury) based on previous studies.^29,73^ The operative sites were evaluated macroscopically to categorize the severity of peritendinous adhesions by using an adhesion scoring system, as previously described.^74,75^ We recruited an additional 24 patients who were treated on an outpatient basis (18 after digit tendon repair and 6 after skin repair [as controls]; 16 men and 8 women) with ages ranging from 24 to 43 years. The patients who underwent digit tendon repair were distributed evenly among the initial, early, and late stages. The total active motion^76,77^ of the injured digit was evaluated. Specimens were frozen immediately after collection at −80 °C for further study. Patients who had tendon rupture caused by rheumatoid arthritis were excluded from this study.

### Mice

The protocols of this study were approved by the Animal Care and Use Committee of The Johns Hopkins University (Baltimore, Maryland, USA) and the Shanghai Sixth People’s Hospital (Shanghai, China) Review Board. All mice were housed in the experimental animal facility of The Johns Hopkins University School of Medicine. *C57BL/6J* (wild-type) mice were obtained from the Jackson Laboratory. Male mice at 10 weeks of age were anesthetized with ketamine and xylazine. After anesthetization, a posterior midline incision was performed in the hind paw, and the FDL tendon was transversally transected and repaired using modified Kessler sutures to create the PAF model as previously described.^54,55^ Then, the FDL was cut at the myotendinous junction to protect the repaired tendon. The wound was closed with 5-0 sutures. The mice could move freely in their cages. Sham surgery followed the same incision procedure, but the deep fascia and tendon were left intact. The sham group was harvested on Day 14 postoperatively.

*LysM-cre* (stock number: 004781) and *TGFß1*^*flox/flox*^ (stock number: 010721) mice were purchased from the Jackson Laboratory. Then, *LysM-cre*::*TGF-β1*^*flox/flox*^ (*TGF-β1*^*–/–*^) mice were generated by crossing *LysM-cre* mice with *TGF-β1*^*flox/flox*^ (*TGF-β1*^*f/f*^) mice. The PAF model (*n* = 8 per group) was established in 10-week-old male *TGF-β1*^*–/–*^ and *TGF-β1*^*f/f*^ mice, which were euthanized after 2, 3, or 4 weeks for further analysis.

*Nestin-creER*^*T2*^ (stock number: 016261) and R26R-EYFP (stock number: 006148) mice were obtained from the Jackson Laboratory, and *TGFβR2* (C57BL/6 background) mice were acquired from the laboratory of H.L. Moses (Vanderbilt University, Nashville, Tennessee, USA).^78^ Subsequently, *Nestin-cre ER*^*T2*^::*TGFβR2*^*flox/flox*^ mice (*TGFβR2*^*f/f*^) were generated by breeding *Nestin-creER*^*T2*^ mice with *TGFβR2*^*flox/flox*^ mice. The PAF model was established in male *TGFβR2*^*–/–*^ and *TGFβR2*^*f/f*^ mice. Beginning one day after the surgery, intraperitoneal injection of 80 mg/kg body weight tamoxifen or vehicle was performed 3 times per week until the mice were euthanized (*n* = 8 per group). Eight male PAF model *Nestin-creER*^*T2*^ male mice injected with tamoxifen were used as controls.

*Nestin-creERT2::R26R-EYFP* mice were generated by breeding *TGFβR2*^*f/f*^ mice with *R26R-EYFP* mice. We also established the PAF model in male *Nestin-creER*^*T2*^*::R26R-EYFP* mice at 10 weeks of age. Beginning on the day after the operation, intraperitoneal injection of 80 mg/kg body weight tamoxifen or vehicle was performed 3 times per week for 4 weeks (*n* = 8 per group). Eight male PAF model *Nestin-creER*^*T2*^ mice were injected with tamoxifen as controls.

For the treatment experiments, a monoclonal TGF-β1 antibody (1D11) was used to neutralize 3 major active TGF-β ligands of TGF-β receptor kinase (TGF-β1, - β2, and - β3).^79^ Eight-week-old male wild-type mice (*n* = 8 per group) were intraperitoneally injected 3 times per week for 1 week from the day of PAF establishment and for 1 week, 2 weeks, or 3 weeks after PAF relative to mice treated with intraperitoneal injection of vehicle for 4 weeks.

### Specimen collection

The mice were euthanized by inhalation of carbon dioxide. Then, the mice were fixed with 4% paraformaldehyde via perfusion through the left ventricle for 5 minutes and stored for 1 day. The feet were collected, immersed in 4% paraformaldehyde, and decalcified in 10% ethylenediaminetetraacetic acid (VWR, 0105) (buffered with phosphate-buffered saline [PBS], pH 7.4) for 1 month. Subsequently, the specimens were embedded in paraffin through a series of ethanol solutions at room temperature or in optimal cutting temperature compound (VWR, 25608-930) after being treated with 20% sucrose (Sigma‒Aldrich, S9378) at −80 °C.

### Histochemistry, immunohistochemistry, and immunofluorescence analysis

Four-μm and 8-μm sagittal sections were cut using a Microm cryostat (for frozen blocks) and a paraffin microtome (for paraffin blocks), respectively. The sections were hydrated in a series alcohol solutions and subsequently subjected to H&E and Masson staining. The orientation of the cellular matrix and the organization of peritendinous adhesions were independently analyzed by two pathologists. Histologic scoring systems and the percentage of the adhesion area relative to the repaired site were used to examine adhesions.^26,75,80^

We performed immunofluorescence staining of PAF sections. Briefly, the sections were rehydrated and washed 3 times with PBS. After blocking endogenous peroxidase activity and nonspecific sites, the tissue sections were incubated with primary antibodies against human/mouse CD68 (Abcam, ab955, 1:200), human/mouse CD206 (Abcam, ab64693, 1:200), human/mouse iNOS (Abcam, ab 15323, 1:200), human CD73 (Abcam, ab175396, 1:100), human CD90 (Abcam, ab81469, 1:100), mouse Nestin (Aves Labs, NES, 1:200), human/mouse α-SMA (Abcam, ab21027, 1:200) and mouse YFP (Abcam, ab290, 1:200), overnight at 4 °C. Subsequently, secondary antibodies were added and incubated with the sections at room temperature for 1 h, and the sections were mounted with DAPI (Life Technologies, P36935). Isotype-matched controls, such as polyclonal rabbit IgG (R&D Systems, AB-105-C), polyclonal goat 511 IgG (R&D Systems, AB-108-C), and monoclonal rat IgG2A (R&D Systems, 54447), were used as negative controls under the same concentrations and conditions. Images were captured with a Zeiss 780 confocal microscope.

We performed immunohistochemical staining of human/mouse collagen I (Abcam, ab34710, 1:200), human/mouse collagen III (Abcam, ab7778, 1:200), human/mouse pSmad2/3 (Santa Cruz Biotechnology, sc-11769, 1:50) and human/mouse pSmad1/5/8 (Cell Signaling Technology, 9516, 1:200). Briefly, the paraffin-embedded specimens were dewaxed, hydrated, and heated to 99 °C for 20 min in Target Retrieval Solution (Dako, S1699) for antigen retrieval. Subsequently, the tissue sections were incubated with primary antibodies overnight at 4 °C. Secondary antibodies were added and incubated with the sections at room temperature for 1 h, and then staining was developed using DAB solution (Dako, K3468), followed by counterstaining with hematoxylin (Sigma‒Aldrich, H9627).

Positively stained cells in 5 random visual fields were quantified and normalized to the number per millimeter in the PAF area. For collagen deposition quantification, we calculated the integral optical density of brown color in 5 random visual fields per stain in each group and normalized them to the integral optical density per millimeter in the PAF area. Analyses were performed using ImageJ software (version 1.48u4).

### Analysis of peritendinous TGF-β1 levels

The concentration of active TGF-β1 in PAF tissues around the repaired tendon was determined by the TGF-β1 ELISA Development kit (human: R&D Systems, DB100B; mouse: R&D Systems, MB100B).

### Assessment of gliding function

The skin of the hind limb that was amputated at the knee level was removed down to the ankle (*n* = 8 per group). The proximal terminal FDL was exposed and secured using cyanoacrylate between 2 pieces of tape.^81,82^ First, the tibia was stabilized by an alligator clip. Then, the FDL was pulled with 0–19 g weights, and associated digital images were captured to investigate the ROM of the metatarsophalangeal joint. Complete flexion was defined according to that of the digits with uninjured tendons loaded with 19 g. Finally, gliding resistance was calculated based on the correspondence of the ROM with loads. Higher gliding resistance indicated more severe adhesion formation.

### Biomechanical testing

To evaluate tendon healing, we calculated the breaking strength and stiffness of the repaired tendon at postoperative Weeks 2, 3, and 4 by using a Dynamic Mechanical Analyzer Q800 (TA Instruments).^68,81^ Specimens were prepared by amputation of the tibia at the ankle. The terminals of the repaired tendon were stabilized to the opposing ends of force gauges. Then, the proximal terminal was pulled in tension at a rate of 30 mm per min until rupture to obtain a force‒displacement curve. The breaking force was documented automatically by the rheometer, while stiffness was calculated as the slope of the linear region of the force‒displacement curve.

### Statistical analysis

SPSS software (version 10.0, IBM Corp, Armonk, New York, USA) was used for all statistical analyses. The data are presented as the mean ± standard deviation. Paired 2-tailed *t* tests were used for comparisons between 2 groups, and 1-way analysis of variance followed by the least significant difference test was used for multiple comparisons between each group. A significant difference was considered when *p* < 0.05. The sample size was not predetermined by statistical methods. The investigators were not blinded to the assignments during the experiment and outcome assessment. All inclusion/exclusion criteria were preestablished, and no samples or animals were excluded from the analysis.

### Study approval

All procedures for the handling of experimental animals were performed according to the policies of John Hopkins after permission was obtained (Baltimore, MD, USA; certificate number SCXK 2003-0003).

## Supplementary information

Supplementray materials

## Data Availability

The data that support the findings of this study are available within the article and Supplementary Files or are available from the corresponding author upon reasonable request.

## References

[1] Weis C (2004). Poly (vinyl alcohol) membranes for adhesion prevention. J. Biomed. Mater. Res. Part B: Appl. Biomater..

[2] Ellis H (1999). Adhesion-related hospital readmissions after abdominal and pelvic surgery: a retrospective cohort study. Lancet..

[3] Shalumon K-T (2018). Multi-functional electrospun antibacterial core-shell nanofibrous membranes for prolonged prevention of post-surgical tendon adhesion and inflammation. Acta Biomater..

[4] Nazifi O, Stuart A-L, Nikkhah D (2020). The use of 5-fluorouracil in the prevention of tendon adhesions: a systematic review. Anim. Model Exp. Med..

[5] Titan A-L (2019). Flexor tendon: development, healing, adhesion formation, and contributing growth factors. Plast. Reconstr. Surg..

[6] Shi X (2019). Prevention of postoperative adhesion reformation by intermittent intrauterine balloon therapy: a randomised controlled trial. BJOG.

[7] van den Beukel B-A (2017). Surgical treatment of adhesion-related chronic abdominal and pelvic pain after gynaecological and general surgery: a systematic review and meta-analysis. Hum. Reprod. Update.

[8] Stapleton L-M (2019). Use of a supramolecular polymeric hydrogel as an effective post-operative pericardial adhesion barrier. Nat. Biomed. Eng..

[9] Feng B (2019). Bioresorbable electrospun gelatin/polycaprolactone nanofibrous membrane as a barrier to prevent cardiac postoperative adhesion. Acta Biomater..

[10] Stommel MWJ (2018). Multicenter observational study of adhesion formation after open-and laparoscopic surgery for colorectal cancer. Ann. Surg..

[11] Fortin C-N, Saed G-M, Diamond M-P (2015). Predisposing factors to post-operative adhesion development. Hum. Reprod. Update.

[12] Ray N-F (1998). Abdominal adhesiolysis: inpatient care and expenditures in the United States in 1994. J. Am. Coll. Surg..

[13] Dy C-J (2012). Complications after flexor tendon repair: a systematic review and meta-analysis. J. Hand Surg..

[14] Loiselle A-E, Kelly M, Hammert W-C (2016). Biological augmentation of flexor tendon repair: a challenging cellular landscape. J. Hand Surg. Am..

[15] Liu C (2019). Biological amnion prevents flexor tendon adhesion in zone II: a controlled, multicentre clinical trial. Biomed. Res. Int..

[16] Nichols AEC, Best K-T, Loiselle A-E (2019). The cellular basis of fibrotic tendon healing: challenges and opportunities. Transl. Res..

[17] Ishiyama N (2010). The prevention of peritendinous adhesions by a phospholipid polymer hydrogel formed in situ by spontaneous intermolecular interactions. Biomaterials.

[18] Civan O (2020). Tenolysis rate after zone 2 flexor tendon repairs. Jt Dis. Relat. Surg..

[19] Azari KK, Meals RA (2005). Flexor tenolysis. Hand Clin..

[20] Graham D-J (2019). The effect of extensor tendon adhesions on finger motion. J. Hand Surg. Am..

[21] Breton, A., Dautel G. Finger flexor tenolysis. Chirurgie de la main, **33 Suppl,** S48–S57 (2014). 10.1016/j.main.2014.07.005 25281402

[22] Klass B-R, Rolfe K-J, Grobbelaar A-O (2009). In vitro flexor tendon cell response to TGF-β1: a gene expression study. J. Hand Surg..

[23] Best K-T (2019). Deletion of NFKB1 enhances canonical NF-kappaB signaling and increases macrophage and myofibroblast content during tendon healing. Sci. Rep..

[24] Kang Y-M (2020). Follistatin mitigates myofibroblast differentiation and collagen synthesis of fibroblasts from scar tissue around injured flexor tendons. Yonsei Med. J..

[25] Jørgensen H-G (2005). Neutralisation of TGFβ or binding of VLA-4 to fibronectin prevents rat tendon adhesion following transection. Cytokine.

[26] Liu S (2012). Biomimetic sheath membrane via electrospinning for antiadhesion of repaired tendon. Biomacromolecules.

[27] Hu C (2013). Long-term drug release from electrospun fibers for in vivo inflammation prevention in the prevention of peritendinous adhesions. Acta Biomater..

[28] Liu S (2013). Prevention of peritendinous adhesions with electrospun ibuprofen-loaded poly(L-lactic acid)-polyethylene glycol fibrous membranes. Tissue Eng. Part A.

[29] Beredjiklian P-K (2003). Biologic aspects of flexor tendon laceration and repair. JBJS.

[30] Kvist M (1985). Fine structural alterations in chronic Achilles paratenonitis in athletes. Pathol.-Res. Pract..

[31] Plikus M-V (2017). Regeneration of fat cells from myofibroblasts during wound healing. Science.

[32] Lee J-H (2017). Erratum to: Specific disruption of Lnk in murine endothelial progenitor cells promotes dermal wound healing via enhanced vasculogenesis, activation of myofibroblasts, and suppression of inflammatory cell recruitment. Stem Cell Res. Ther..

[33] Wong J-KF (2009). The cellular biology of flexor tendon adhesion formation: an old problem in a new paradigm. Am. J. Pathol..

[34] Li M (2019). Regulatory effects of dermal papillary pluripotent stem cells on polarization of macrophages from M1 to M2 phenotype in vitro. Transplant. Immunol..

[35] Kimura, T. et al. Polarization of M2 macrophages requires Lamtor1 that integrates cytokine and amino-acid signals. *Nat. Commun.* 713130 (2016). 10.1038/ncomms13130 PMC5064021 27731330

[36] Moganti K (2017). Hyperglycemia induces mixed M1/M2 cytokine profile in primary human monocyte-derived macrophages. Immunobiology.

[37] Khan J, Sharma PK, Mukhopadhaya A (2015). Vibrio cholerae porin OmpU mediates M1-polarization of macrophages/monocytes via TLR1/TLR2 activation. Immunobiology.

[38] Wang J (2019). GTS-21 reduces inflammation in acute lung injury by regulating M1 polarization and function of alveolar macrophages. Shock.

[39] Hedl M (2019). IRF5 is required for bacterial clearance in human M1-polarized macrophages, and IRF5 immune-mediated disease risk variants modulate this outcome. J. Immunol..

[40] Braune J (2017). IL-6 regulates M2 polarization and local proliferation of adipose tissue macrophages in obesity. J. Immunol..

[41] Kimura T (2016). Polarization of M2 macrophages requires Lamtor1 that integrates cytokine and amino-acid signals. Nat. Commun..

[42] Oishi S (2016). M2 polarization of murine peritoneal macrophages induces regulatory cytokine production and suppresses T‐cell proliferation. Immunology.

[43] Wang X (2018). Inhibition of overactive TGF-β attenuates progression of heterotopic ossification in mice. Nat. Commun..

[44] Chang J (2000). Studies in flexor tendon wound healing: neutralizing antibody to TGF-β1 increases postoperative range of motion. Plast. Reconstruct. Surg..

[45] Tang Y (2009). TGF-β1–induced migration of bone mesenchymal stem cells couples bone resorption with formation. Nat. Med..

[46] Blobe G-C, Schiemann W-P, Lodish H-F (2000). Role of transforming growth factor β in human disease. N. Engl. J. Med..

[47] Wang X (2018). Aberrant TGF-β activation in bone tendon insertion induces enthesopathy-like disease. J. Clin. Investig..

[48] Bruno V (2018). Effects of low molecular weight heparin on the polarization and cytokine profile of macrophages and T helper cells in vitro. Sci Rep..

[49] Han C-H (2017). Polarization of macrophages in the blood after decompression in mice. Med. Gas Res..

[50] Consentius C (2018). In situ detection of CD73+ CD90+ CD105+ lineage: Mesenchymal stromal cells in human placenta and bone marrow specimens by chipcytometry. Cytom. Part A.

[51] Pittenger M-F (1999). Multilineage potential of adult human mesenchymal stem cells. Science.

[52] Edanami N (2017). Characterization of dental pulp myofibroblasts in rat molars after pulpotomy. J. Endod..

[53] Artlett C-M (2011). The inflammasome activating caspase 1 mediates fibrosis and myofibroblast differentiation in systemic sclerosis. Arthritis Rheum..

[54] Ackerman JE, Loiselle A-E (2016). Murine flexor tendon injury and repair surgery. J. Vis. Exp..

[55] Fernando M-R, Giembycz M-A, McKay D-M (2016). Bidirectional crosstalk via IL-6, PGE2 and PGD2 between murine myofibroblasts and alternatively activated macrophages enhances anti-inflammatory phenotype in both cells. Br. J. Pharmacol..

[56] Groszer M (2001). Negative regulation of neural stem/progenitor cell proliferation by the Pten tumor suppressor gene in vivo. Science.

[57] Khan J-A (2016). Fetal liver hematopoietic stem cell niches associate with portal vessels. Science.

[58] Mendez-Ferrer S (2010). Mesenchymal and haematopoietic stem cells form a unique bone marrow niche. Nature.

[59] Ackerman J-E (2017). Deletion of EP4 in S100a4-lineage cells reduces scar tissue formation during early but not later stages of tendon healing. Sci. Rep..

[60] Geary M-B (2015). Systemic EP4 inhibition increases adhesion formation in a murine model of flexor tendon repair. PLoS One.

[61] Clausen B-E (1999). Conditional gene targeting in macrophages and granulocytes using LysMcre mice. Transgenic Res..

[62] Wan M (2012). Injury‐activated transforming growth factor β controls mobilization of mesenchymal stem cells for tissue remodeling. Stem Cells.

[63] Loiselle A-E (2012). Bone marrow-derived matrix metalloproteinase-9 is associated with fibrous adhesion formation after murine flexor tendon injury. PloS One.

[64] Dou C (2022). Sialylation of TLR2 initiates osteoclast fusion. Bone Res..

[65] Dakin S-G (2012). Macrophage sub-populations and the lipoxin A4 receptor implicate active inflammation during equine tendon repair. PLoS One.

[66] Tang P-M, Nikolic-Paterson D-J, Lan H-Y (2019). Macrophages: versatile players in renal inflammation and fibrosis. Nat. Rev. Nephrol..

[67] Nathan C (2012). Secretory products of macrophages: twenty-five years on. J. Clin. Investis..

[68] Hui W-W (2018). Salmonella enterica Serovar Typhimurium Alters the Extracellular Proteome of Macrophages and Leads to the Production of Proinflammatory Exosomes. Infect. Immun..

[69] Talsma D-T (2018). Endothelial heparan sulfate deficiency reduces inflammation and fibrosis in murine diabetic nephropathy. Lab. Investig..

[70] Katzel E-B (2011). Impact of Smad3 loss of function on scarring and adhesion formation during tendon healing. J. Orthop. Res..

[71] Kakudo N (2012). Effects of transforming growth factor-beta1 on cell motility, collagen gel contraction, myofibroblastic differentiation, and extracellular matrix expression of human adipose-derived stem cell. Hum. Cell.

[72] Yang L (2012). Bone marrow-derived mesenchymal stem cells differentiate to hepatic myofibroblasts by transforming growth factor-beta1 via sphingosine kinase/sphingosine 1-phosphate (S1P)/S1P receptor axis. Am. J. Pathol..

[73] Gelberman R-H (2017). Combined administration of ASCs and BMP-12 promotes an M2 macrophage phenotype and enhances tendon healing. Clin. Orthop. Relat. Res..

[74] Cashman J (2004). Camptothecin-loaded films for the prevention of postsurgical adhesions. Inflamm. Res..

[75] Liu S (2013). Tendon healing and anti-adhesion properties of electrospun fibrous membranes containing bFGF loaded nanoparticles. Biomaterials.

[76] Lee K-H (2018). Clinical results of autogenous palmaris longus tendon graft for ruptures of multiple extensors in rheumatoid hands. J. Hand Surg. Am..

[77] Cavadas P-C, Thione A, Rubi C (2016). Hand amputations at the radiocarpal level with proximal neuromuscular avulsion. J. Hand Surg. Am..

[78] Chytil A (2002). Conditional inactivation of the TGF-beta type II receptor using Cre:Lox. Genesis.

[79] Dasch J-R (1989). Monoclonal antibodies recognizing transforming growth factor-beta. Bioactivity neutralization and transforming growth factor beta 2 affinity purification. J. Immunol..

[80] Gudemez E (2002). Chondroitin sulfate-coated polyhydroxyethyl methacrylate membrane prevents adhesion in full-thickness tendon tears of rabbits. J. Hand Surg. Am..

[81] Loiselle A-E (2009). Remodeling of murine intrasynovial tendon adhesions following injury: MMP and neotendon gene expression. J. Orthop. Res..

[82] Hasslund S (2008). Adhesions in a murine flexor tendon graft model: autograft versus allograft reconstruction. J. Orthop. Res..

