# Supplementary material for: Neutralization of excessive levels of active TGF-β1 reduces MSC recruitment and differentiation to mitigate peritendinous adhesion
Source: Bone Res. 2023 May 8;11:24. doi: 10.1038/s41413-023-00252-1 (PMC10167238; doi:10.1038/s41413-023-00252-1)
Supplement: Supplementary file 1 — Supplementray materials [file 41413_2023_252_MOESM1_ESM.doc]

**
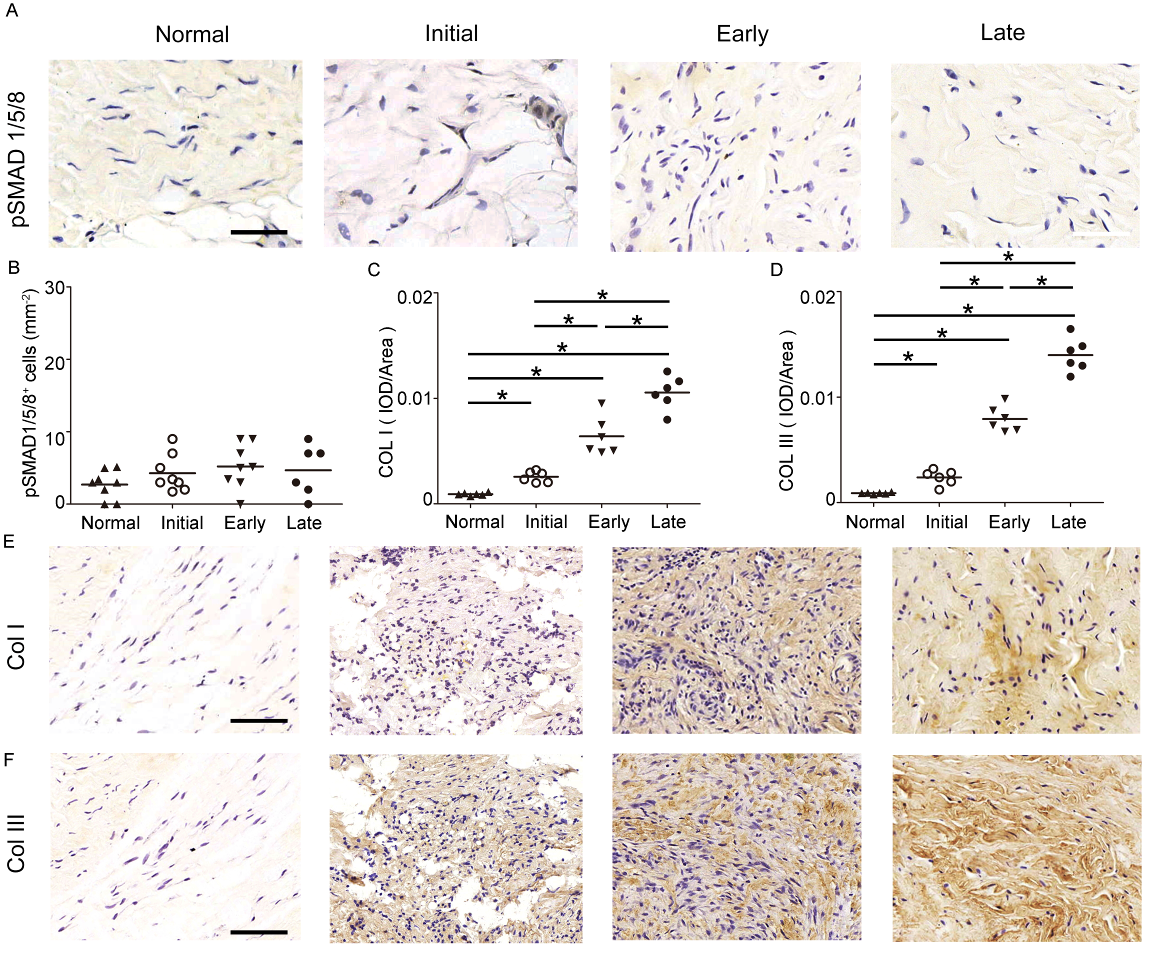
**

**Supplementary Figure 1. Elevated collagen I and III deposition in human PAF tissues.** Immunohistological staining (A) and quantitative analysis (B) of the number of pSmad1/5/8^+^ cells (brown) per PAF tissue area (mm^2^). Scale bar, 400μm. Quantitative analysis of collagen (Col) I (brown) (C) and Col III (brown) (D) deposition in PAF tissues. Immunohistological staining of Col I (E) and Col III (F) deposition in PAF tissues. Normal, normal surrounding tissues around tendons; Initial, Early, and Later indicates the time after tendon injury at day 1–2, day 8–10, or week 12–14, respectively. Scale bar, 200μm.* indicates P < 0.05.


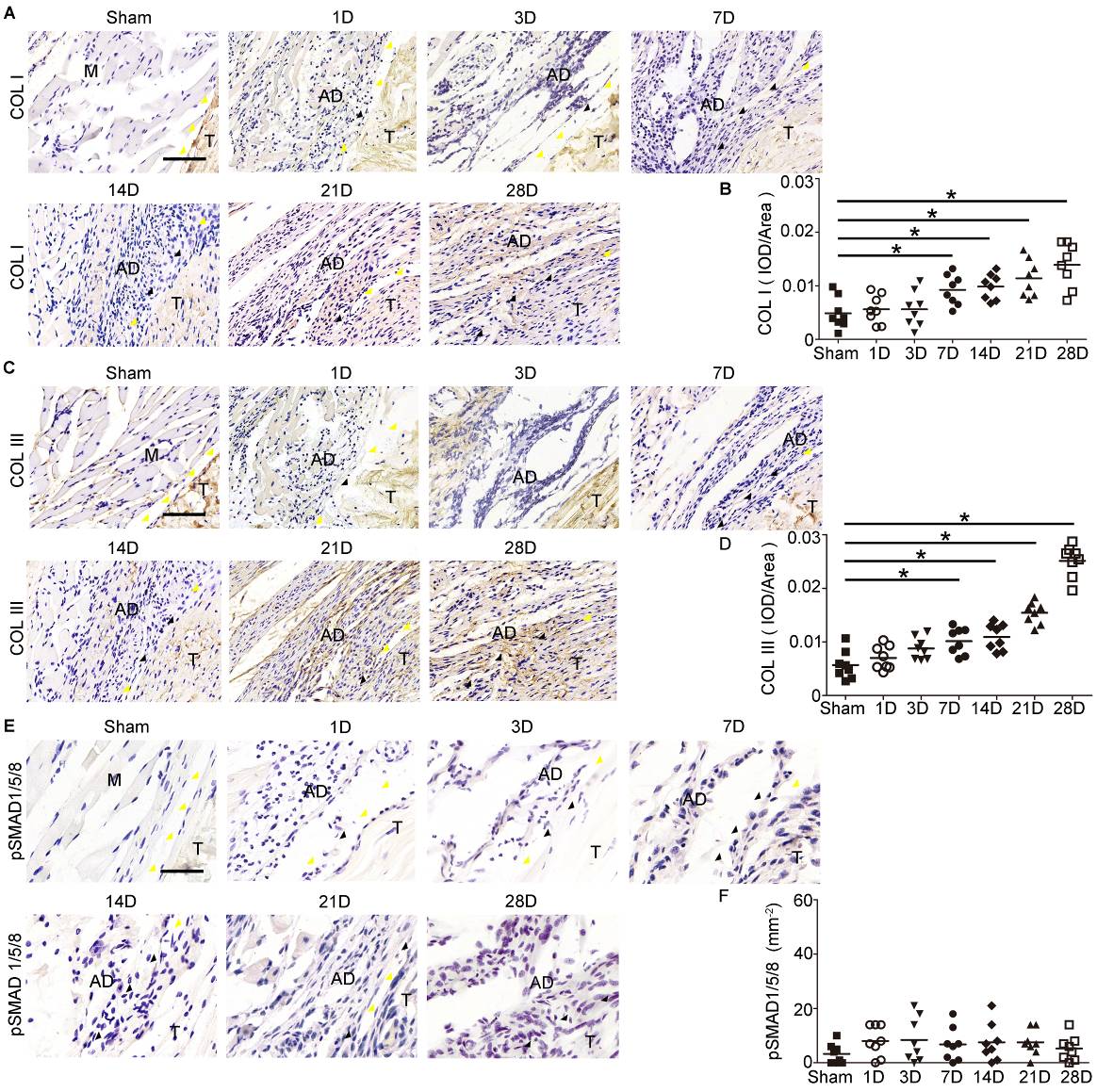


**Supplementary Figure 2. Elevated Collagen I and III deposition in mouse PAF tissues.** Immunohistological staining and quantitative analysis of collagen (Col) I (brown) (A) and Col III (brown) (B) deposition in PAF tissues, and immunohistological staining of Col I (brown) (C) and Col III (brown) (D) deposition in PAF tissues at postoperative 1D, 3D, 7D, 14D, 21D, and 28D. Scale bar, 200μm.Immunohistological staining (E) and quantitative analysis (F) of the number of pSmad1/5/8^+^ cells (brown) per PAF tissue area (mm^2^) on postoperative day 1 (1D), 3 (3D), 7 (7D), 14 (14D), 21 (21D), and 28 (28D). Scale bar, 400 μm. Yellow arrowheads show space between tendon and its surrounding tissues. Black arrowheads show space occupied by adhesion tissues.AD, adhesion tissues; M, muscle; T, tendon. * indicates P < 0.05.

**
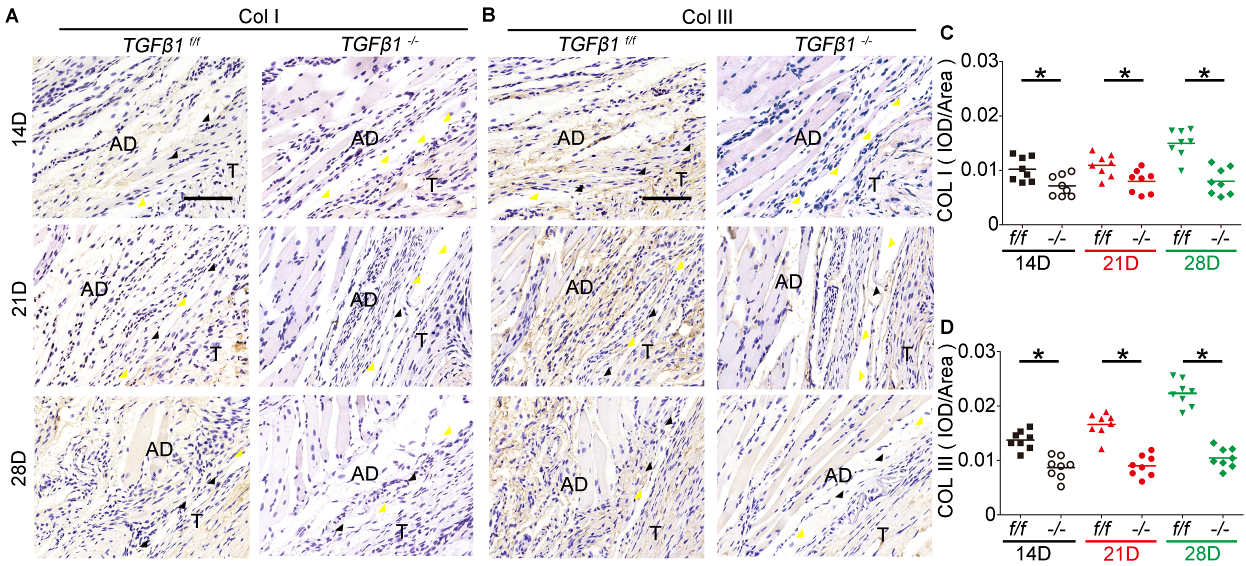
**

**Supplementary Figure 3. Knockout of TGF-β1 in Lysm^+^ cells reduces collagen I and III deposition in mouse PAF tissues.** Immunohistological staining of collagen (Col) I (brown) (A) and Col III (brown) (B) deposition in PAF tissues on postoperative day 14 (14D), 21 (21D), and 28 (28D). Quantitative analysis of Col I (brown) (C) and Col III (brown) (D) deposition in PAF tissues. Scale bar, 200μm. AD, adhesion tissues; M, muscle; T, tendon. Yellow arrowhead shows space between tendon and its surrounding tissues. Black arrowheads show space occupied by adhesion tissues. * indicates P < 0.05.


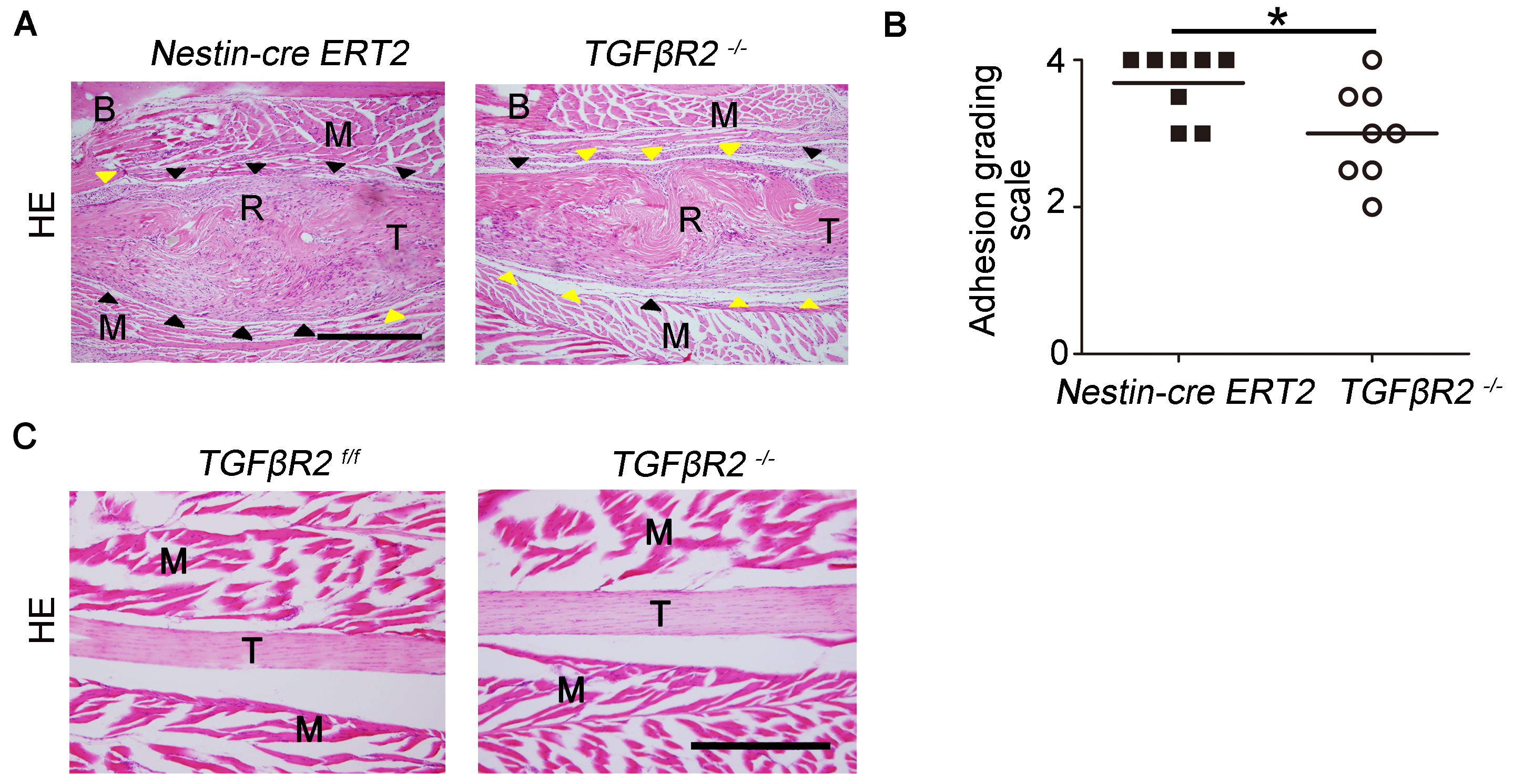


**Supplementary Figure 4. Comparison of adhesion between *Nestin-creER^T2^* and *TGFβR2^–/–^mice* with injection of tamoxifen and α-SMA^+^ YFP^+^ cells between *Nestin-creER^T2^::R26R-EYFP* mice with or without injection of tamoxifen.** H&E staining (A) and histological adhesion scores (B) of PAF tissues between *Nestin-creER^T2^* and *TGFβR2^–/–^* mice with injection of tamoxifen (TM) on postoperative day 28 (28D). The Sham groups of TGF-βR2^–/–^ and TGF-βR2^f/f^ mice (C). Yellow arrowheads show space between tendon and its surrounding tissues. Black arrowheads show space occupied by adhesion tissues. Scale bar, 1mm.B, bone; M, muscle; R, repaired site; T, tendon. * indicates P < 0.05.

**
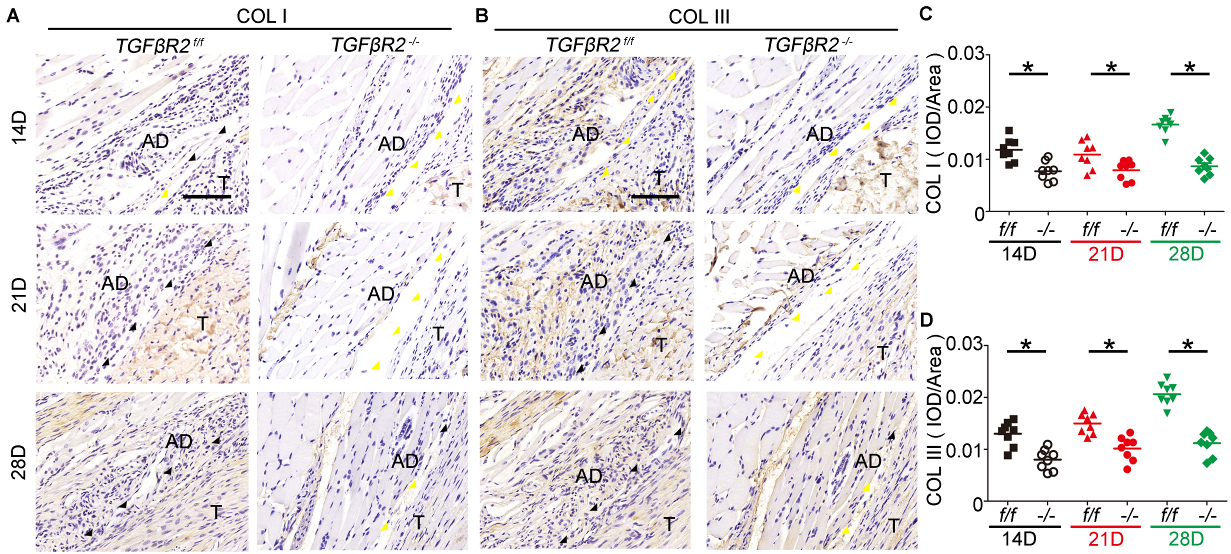
**

**Supplementary Figure 5. Deletion of TGFβR2 in Nestin^+^ cells reduced collagen I and III deposition in PAF tissues.** Immunohistological staining of collagen (Col) I (brown) (A) and Col III (brown) (B) deposition in PAF tissues. Quantitative analysis of Col I (brown) (C) and Col III (brown) (D) deposition in PAF tissues. Scale bar, 200 μm. AD, adhesion tissues; M, muscle; T, tendon. Yellow arrowheads show space between tendon and its surrounding tissues. Black arrowheads show space occupied by adhesion tissues. * indicates P < 0.05.

**
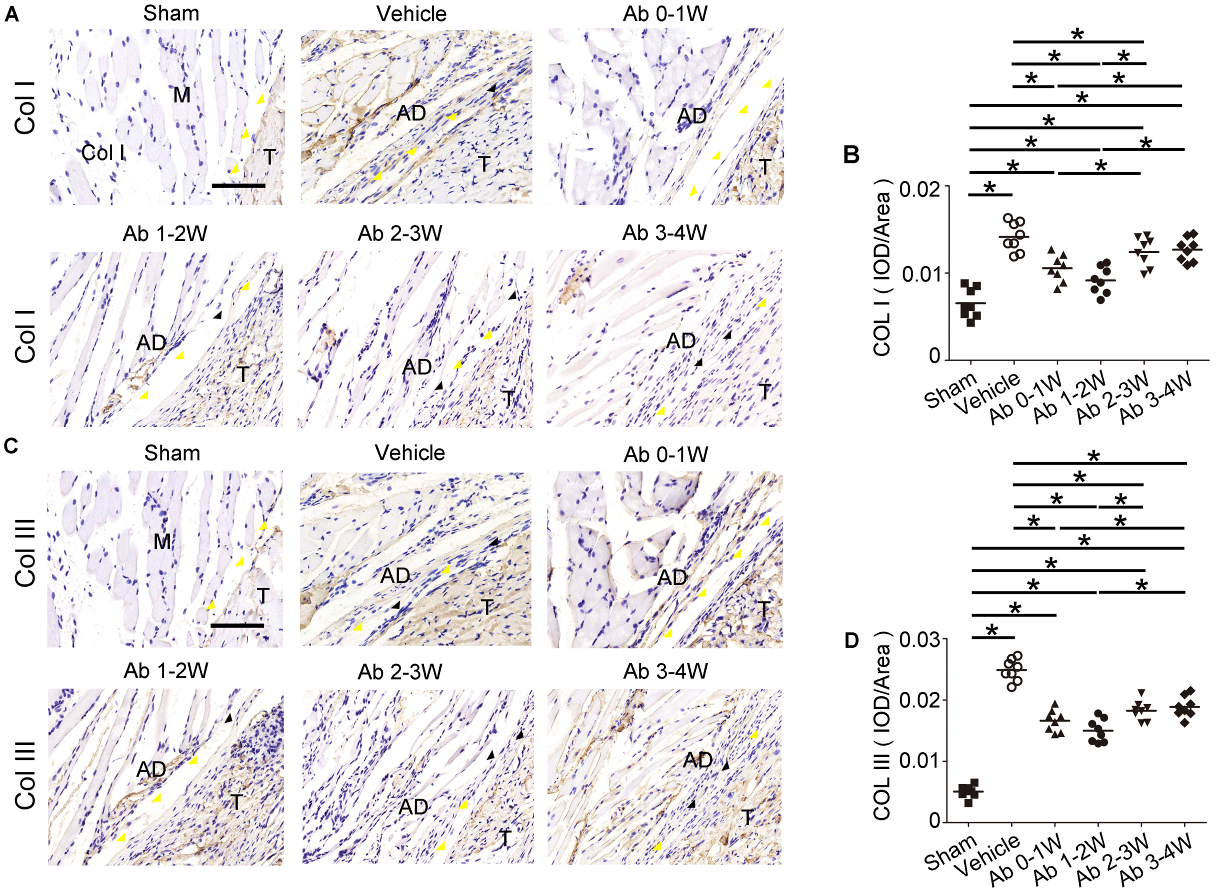
**

**Supplementary Figure 6. Systemic injection of TGF-β1 neutralizing antibody reduced collagen I and collagen III in PAF tissues.** Mice were treated with vehicle3 times a week for 4weeks or 5mg/kg body weight of the TGF-β1 neutralizing antibody 1D11 three times a week from postoperative day 1 (Ab 0–1W), week 1 (Ab 1–2W), week 2 (Ab 2–3W), or week 3 (Ab 3–4W) for 1 week until euthanasia at week 4. Immunohistological staining (A) and quantitative analysis (B) of collagen (Col) I (brown) deposition in PAF tissues. Immunohistological staining (C) and quantitative analysis (D) of Col III deposition in PAF tissues. Scale bar, 200μm. AD, adhesion tissues; M, muscle; T, tendon. Yellow arrowheads show space between tendon and its surrounding tissues. Black arrowheads show space occupied by adhesion tissues. * indicates P < 0.05.

**
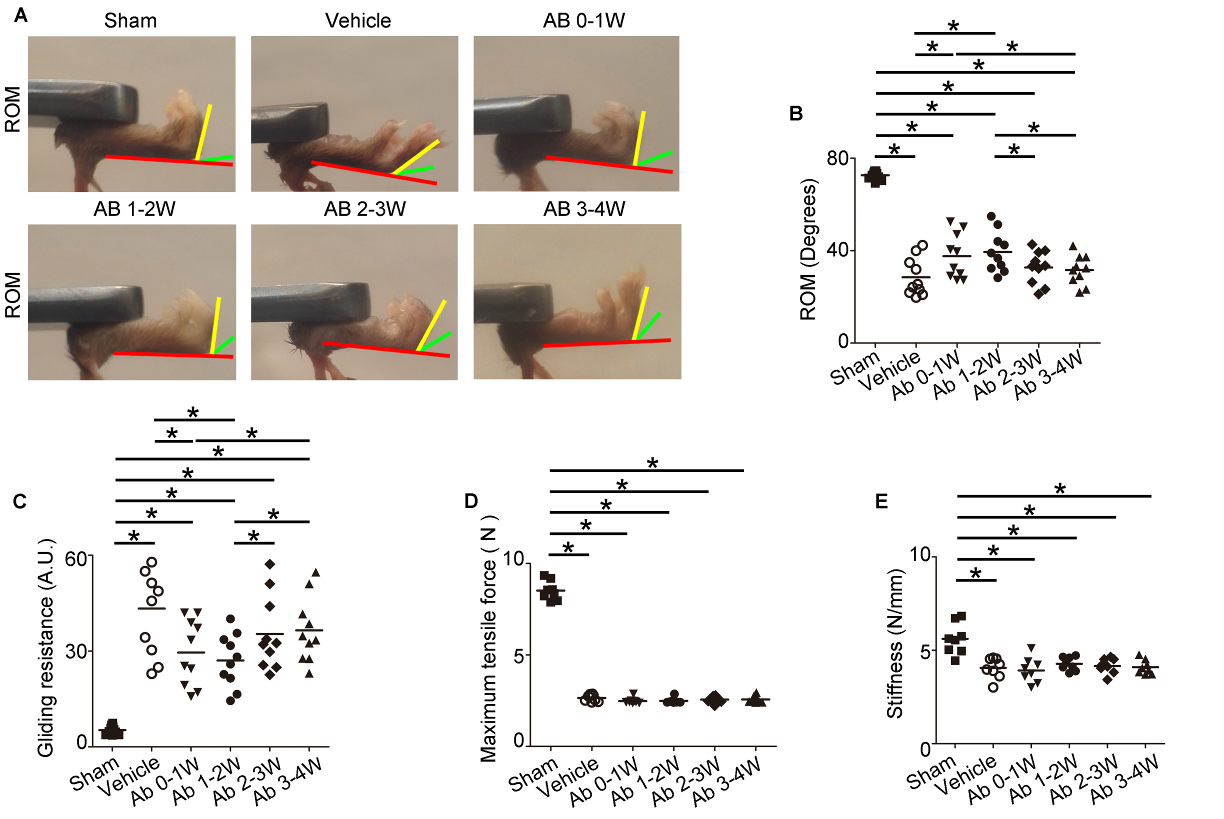
**

**Supplementary Figure 7. Systemic injection of TGF-β1 neutralizing antibody reduced collagen I and collagen III in PAF tissues.** Mice were treated with vehicle3 times a week for 4weeks or 5mg/kg body weight of the TGF-β1 neutralizing antibody 1D11 3 times a week from postoperative day 1 (Ab 0–1W), week 1 (Ab 1–2), week 2 (Ab 2–3W), or week 3 (Ab 3–4W) for 1 week until euthanasia at week 4. Investigation (A) and quantitative analysis (B) of range of motion on postoperative day 28 (28D). Quantitative analysis of gliding resistance (C), maximum tensile force (D), and stiffness (E) of repaired tendons. * indicates P < 0.05.
